# Defining the Role of the MHC in Autoimmunity: A Review and Pooled Analysis

**DOI:** 10.1371/journal.pgen.1000024

**Published:** 2008-04-25

**Authors:** Michelle M. A. Fernando, Christine R. Stevens, Emily C. Walsh, Philip L. De Jager, Philippe Goyette, Robert M. Plenge, Timothy J. Vyse, John D. Rioux

**Affiliations:** 1 Section of Molecular Genetics and Rheumatology, Faculty of Medicine, Imperial College London, London, United Kingdom; 2 Program in Medical and Population Genetics, Broad Institute, Massachusetts Institute of Technology and Harvard University, Cambridge, Massachusetts, United States of America; 3 Department of Neurology, Center for Neurologic Diseases, Brigham and Women's Hospital and Harvard Medical School, Boston, Massachusetts, United States of America; 4 Harvard Medical School/Partners Healthcare Center for Genetics and Genomics, Boston, Massachusetts, United States of America; 5 Université de Montréal, Montréal Heart Institute, Montréal, Québec, Canada; 6 Harvard Medical School, Division of Rheumatology, Allergy and Immunology, Boston, Massachusetts, United States of America; University College London, United Kingdom

## Abstract

The major histocompatibility complex (MHC) is one of the most extensively studied regions in the human genome because of the association of variants at this locus with autoimmune, infectious, and inflammatory diseases. However, identification of causal variants within the MHC for the majority of these diseases has remained difficult due to the great variability and extensive linkage disequilibrium (LD) that exists among alleles throughout this locus, coupled with inadequate study design whereby only a limited subset of about 20 from a total of approximately 250 genes have been studied in small cohorts of predominantly European origin. We have performed a review and pooled analysis of the past 30 years of research on the role of the MHC in six genetically complex disease traits – multiple sclerosis (MS), type 1 diabetes (T1D), systemic lupus erythematosus (SLE), ulcerative colitis (UC), Crohn's disease (CD), and rheumatoid arthritis (RA) – in order to consolidate and evaluate the current literature regarding MHC genetics in these common autoimmune and inflammatory diseases. We corroborate established MHC disease associations and identify predisposing variants that previously have not been appreciated. Furthermore, we find a number of interesting commonalities and differences across diseases that implicate both general and disease-specific pathogenetic mechanisms in autoimmunity.

## Introduction

The major histocompatibility complex (MHC), located on the short arm of Chromosome 6, is one of the most extensively studied regions in the human genome because of the contribution of multiple variants at this locus in autoimmune, infectious, and inflammatory diseases and in transplantation. Historically, the murine MHC locus, *H2*, was identified and subsequently named for its role in histocompatibility almost 60 years ago by George Snell [1]. Shortly afterward, Jean Dausset recognized the human MHC, or human leukocyte antigen (HLA) region, so named because Dausset originally demonstrated MHC antigens on the surface of white blood cells [2]. Subsequently, Baruj Benacerraf described the importance of these antigens in the immune response [3]. The seminal work of Snell, Dausset, and Benacerraf garnered them the 1980 Nobel Prize for Medicine.

The classical MHC encompasses approximately 3.6 megabasepairs (Mb) on 6p21.3 and is divided into three subregions: the telomeric class I, class III, and the centromeric class II regions. The concept of the extended MHC (xMHC), spanning about 7.6 Mb of the genome, has been recently established by the finding that linkage disequilibrium (LD) and MHC-related genes exist outside the classically defined locus [4]. Of the 421 genes within the xMHC, 60% are expressed and approximately 22% have putative immunoregulatory function. The five subregions of the xMHC comprise the extended class I subregion, classical class I, classical class III, classical class II, and the extended class II subregions [4].

The MHC was first associated with disease in 1967 when HLA-B antigens were found at increased frequency in patients with Hodgkin's lymphoma [5]. Since then, variation within the MHC has been found to be associated with almost every autoimmune disease, as well as several infectious and inflammatory diseases. However, because of the extensive LD that exists among alleles throughout this locus, the causal MHC variant(s) have remained elusive for the great majority of diseases.

Nonrandom association (or LD) in the inheritance of alleles at multiple loci within the MHC was demonstrated as early as 1968 [6]. Upon determination of the physical size of the region, it appeared that LD extended more than 2 Mb in some cases, but not all. This differs from the LD pattern reported for other regions in the genome where strong LD exists in segments of approximately 22 kilobases (kb) [7]. However, closer inspection reveals that the “micro”-structure of LD is similar for the MHC [8],[9]. What appears to be different about a subset of MHC haplotypes is that there is a higher amount of LD observed *between* segments of strong LD. Such tight segment-to-segment LD can pose an important obstacle in MHC research: if one identifies a disease association with a variant in the region, it may not be possible to determine whether the variant is causal or whether its association simply reflects LD with the true causal variation. Several studies of the region to date have suffered from this caveat.

A further complication in the identification of disease-causing variants at the MHC is the great variability exhibited by some of the genes within the MHC (such as the classical class I genes, *HLA-A*, *-B,* and *-C* and the classical class II genes, *HLA-DRB1*, *-DQA1,* and *-DQB1*), which require typing strategies that are both labor- and time-intensive; indeed *HLA-B* is the most polymorphic gene known in the human genome.

The mechanisms underlying MHC association in autoimmune disease are not clearly understood. One long-held view suggests a breakdown in immunological tolerance to self-antigens through aberrant class II presentation of self or foreign peptides to autoreactive T lymphocytes. Thus, it seems likely that specific MHC class II alleles determine the targeting of particular autoantigens resulting in disease-specific associations.

For the reasons outlined above, most published studies of MHC disease association to date have been restricted to small cohorts, each testing a limited number of variants using a variety of typing methodologies. This problem has resulted in a literature base that can be complex and at times conflicting. We have therefore examined the past 30 years of research regarding MHC genetics in multiple autoimmune and inflammatory diseases using two different approaches—(1) a review of published data and (2) a pooled analysis of case–control association studies across the region—in order to consolidate and evaluate the current literature base. We chose to investigate six genetically complex disease traits: multiple sclerosis (MS), type 1 diabetes (T1D), systemic lupus erythematosus (SLE), ulcerative colitis (UC), Crohn's disease (CD), and rheumatoid arthritis (RA). By relying on combined data, pooled analyses provide greater power for detecting disease-associated variants, so may be helpful in corroborating or refuting previous findings and establishing additional associations. Specifically, we performed PubMed literature searches and identified references from review sources to create a list of case–control association studies across the region for each disease up to and including September 2005 (see Table S1 for all disease-specific studies included in the pooled analysis and Text S1 for further search details). Only variants for which there were three independent studies examining more than 50 cases each were included in the final analysis. Phenotype and allele frequency data were included but analyzed separately in this study. In total approximately 390 studies were included across all diseases.

**Figure 1:**
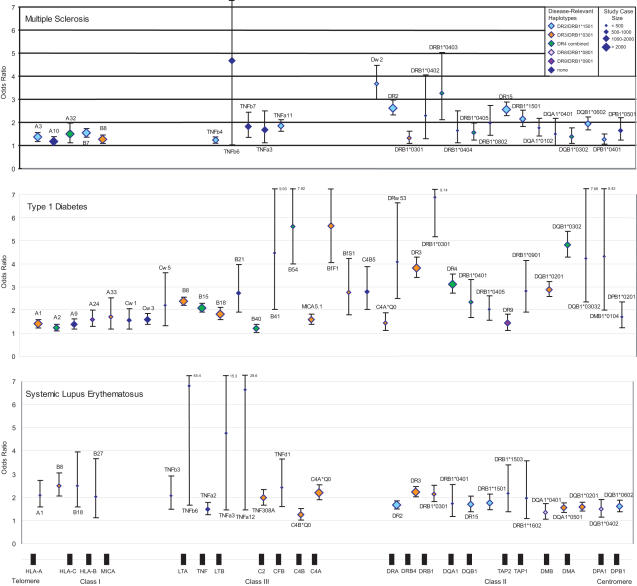
MHC susceptibility alleles identified by pooled analysis: Multiple sclerosis, type 1 diabetes, and systemic lupus erythematosus. Susceptibility is defined as a lower CI greater than 1.0. Shown are odds ratios with 95% CIs for MS (upper graph), T1D (middle graph), and SLE (lower graph). Beneath is a schematic representation of MHC class I, class III, and class II genes in genomic order but not to scale. Diamond size represents total number of cases included in pooled analysis for each allele. Diamond color reflects different disease-relevant ancestral haplotypes.

**Figure 2:**
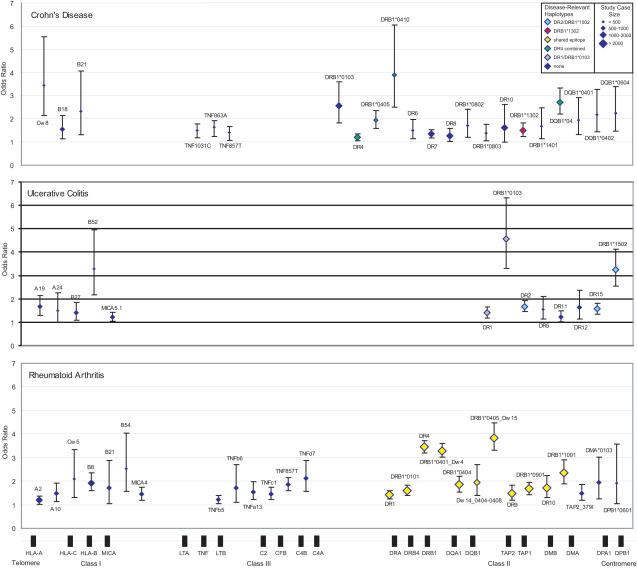
MHC susceptibility alleles identified by pooled analysis: Crohn's disease, ulcerative colitis, and rheumatoid arthritis. Susceptibility is defined as a lower CI greater than 1.0. Shown are odds ratios with 95% CIs for CD (upper graph), UC (middle graph), and RA (lower graph). Beneath is a schematic representation of MHC class I, class III, and class II genes in genomic order but not to scale. Diamond size represents total number of cases included in pooled analysis for each allele. Diamond color reflects different disease-relevant ancestral haplotypes except for the shared epitope alleles in RA.

Odds ratios (ORs) and 95% confidence intervals (CIs) were calculated for each serological, mixed lymphocyte reaction, or molecular specificity (see Text S1). These data were collated (Table S2) and used to create Figures 1 and 2. The extended LD observed at the MHC does not allow differentiation between allelic and haplotypic association in the current pooled analysis. Thus, as it is vital to consider LD when interpreting association results at the MHC, our figures display statistically significant variants on the basis of “ancestral,” also known as “conserved extended,” haplotypes [10]–[12].

The following text is subdivided by disease; for each we provide a disease-specific review of the current literature on MHC genetics and then detail the results of the pooled analysis (illustrated in Figures 1 and 2) with respect to the variants found to confer risk to disease (defined as a lower limit CI >1.0). A more comprehensive account of MHC association for each disease can be found in Datasets S1, S2, S3, S4, and S5. We also provide a brief explanation of HLA nomenclature and typing methodologies (with a mapping of serotypes to genotypes) because of the confusion that often surrounds these topics (Text S2 and Table S3).

## Multiple Sclerosis

MS (Online Mendelian Inheritance in Man [http://www.ncbi.nlm.nih.gov/sites/entrezdbomim] accession number [MIM] 126200) is a chronic inflammatory demyelinating disorder of the central nervous system (reviewed in [13],[14]). The only region of the genome that has shown consistent evidence of linkage and association with MS is the MHC (reviewed in [15]). The association of the *HLA-DR2* haplotype with MS was first noted in 1972 [16] and remains one of the most reproduced findings in MHC genetics. Due to strong LD across the associated haplotype, it remains unclear whether the primary driver for the association of the HLA locus to MS is the HLA-DQB1*0602 allele or the HLA-DRB1*1501 allele (reviewed in [15]). However, evidence is mounting that HLA-DRB1*1501 and the closely related HLA-DRB1*1503 allele are more strongly associated with MS in African-American [17] and possibly European populations (PLD, unpublished data) when compared to HLA-DQB1*0602. In addition, alleles within the MHC class I region show suggestive evidence of association independent of the DRB1*1501–DQB1*0602 haplotype, and include HLA-A*0201 [18], *HLA-A3* [15],[18] and *HLA-Cw05* [19] as well as the HLA C1_3_2*354 microsatellite allele, although the latter is in strong LD with *HLA-DR3* [20].

There is support for allelic heterogeneity within the *HLA-DRB1* gene, particularly in non-European MS populations [19],[21]. The best evidence for a second *HLA-DRB1* risk allele in MS probably lies with HLA-DRB1*03 (*DR3*) [19],[22],[23], although HLA-DRB1*0103 and *HLA-DR4* alleles may also increase disease risk (reviewed in [13]) [19], [24]–[26].

As expected, our pooled analysis highlights the preeminent role of the extended haplotype defined by HLA-DRB1*1501 in MS (Figure 1, top). Two other ancestral haplotypes containing *HLA-DR3* and *HLA-DR4* also appear to play a role in MS susceptibility, although the effect of these haplotypes on disease is more modest than that of the *HLA-DR2* haplotypes. In comparison to the HLA-DRB1*1501 analysis, it is less clear whether the HLA-DRB1*0301 allele is primarily driving the association or whether one of the alleles of *tumor necrosis factor* (*TNF*), for example, could be in stronger LD with a risk allele. On the other hand, the *HLA-DR4* haplotypes seem to display their strongest association with their *HLA-DRB1* alleles: four different *HLA-DR4* alleles—*0402, 0403, 0404,* and *0405*—display significant association in our analysis. While the population risk of *HLA-DR4* haplotypes on MS susceptibility may be relatively small, those rare individuals bearing these alleles may have a large increase in disease risk. In MS, our pooled analysis highlights the class II gene *HLA-DRB1* as the primary candidate for MS susceptibility at the MHC, with a number of different alleles contributing to disease risk (see Dataset S1 for further information).

## Type 1 Diabetes

T1D (MIM 222100) is a chronic autoimmune disease characterized by T cell-mediated destruction of pancreatic islet beta cells, resulting in irreversible insulin deficiency and long-term dysfunction of several organs and tissues. There is no doubt that the major genetic contribution to T1D susceptibility arises from the MHC [27], accounting for approximately 50% of the total genetic contribution to disease [28]. To date most evidence supports a role for variation at *HLA-DQ* as the major disease-predisposing locus [29],[30].

The predominant role of DRB1*04–DQA1*0301–DQB1*0302 and DRB1*03–DQA1*0501–DQB1*0201 haplotypes in susceptibility to T1D in European populations is borne out by several studies [31]. Heterozygosity for both risk haplotypes confers the greatest known genetic risk for T1D [31]. The formation of specific *trans* DQ dimers by transcomplementation between *HLA-DQA1* and *HLA-DQB1* alleles on homologous chromosomes (DQA1*0301/DQB1*0201 and DQA1*0501/DQB1*0302) may be responsible for the increase in heterozygote risk [27],[32],[33]. *HLA-DR4* (DRB1*0405–DQB1*0401) and *HLA-DR9* (DRB1*0901–DQB1*0303) have shown association with T1D in Japanese and Korean populations [34]. The low frequency of the disease-associated *HLA-DR3* and *HLA-DR4* haplotypes may contribute to the reduced incidence of T1D in these non-European populations [31].

The nature of the *HLA-DR* association in T1D remains unclear. LD with *HLA-DQ* alleles may account for some of the association [27], while certain *DRB1* alleles may also modify the risk present at the *DQ* locus. More recent studies, which require validation, suggest a role for a non-HLA locus, telomeric of class I [35], as well as polymorphism within *HLA-DPB1* [36],[37] in susceptibility to T1D.

Overall, studies to date suggest that both *HLA-DR* and *HLA-DQ* genes are important in determining disease risk, but the effects of individual alleles may be modified by the haplotypes on which they are carried [31]. There appears to be a hierarchy of risk alleles from the strongly protective HLA-DQB1*0602 to the highly predisposing HLA-DQB1*0302. Such a spectrum of risk is also borne out by TDT (transmission disequilibrium test) analysis showing that each *HLA-DR/HLA-DQ* haplotype has its own individual disease risk, which may result from transcomplementation and other haplotypic effects.

Our pooled analysis confirms association with *HLA-DR3*, *HLA-DR4* and *HLA-DR9*-containing haplotypes in T1D (Figure 1, middle). The *HLA-DR9*/HLA-DRB1*0901 associations we observe arise from non-European cohorts only and concur with the published literature. With regard to *HLA-DPB1*, we find association with HLA-DPB1*0201, which maps to both disease-associated and unrelated haplotypes. A number of other, mainly class I alleles also show evidence for disease predisposition (see Dataset S2) and warrant further investigation.

## Systemic Lupus Erythematosus

SLE (MIM 152700), or lupus, is the prototypic, multisystem autoimmune disease primarily affecting women of child-bearing age. Recent genome-wide association scans and a meta-analysis of linkage screens confirm the MHC as the greatest genetic risk factor in lupus susceptibility ([38]; TJV, unpublished data). However, the precise contribution attributable to the MHC with respect to overall genetic risk remains to be determined.

The most consistent HLA associations with SLE reside with the class II alleles, *HLA-DR3* (DRB1*0301) and *HLA-DR2* (DRB1*1501) and their respective haplotypes in predominantly white populations [39]. Studies in nonwhite populations have shown inconsistent results [40]–[44]. Inherited (and acquired) deficiencies of the early classical complement components, C2, C4A, and C4B, encoded within the class III region, are associated with the development of lupus [45]. In particular, *C4A* and *C4B* null alleles, which result in partial C4 deficiency, show association with disease [46],[47]. However, these alleles are in strong LD with specific ancestral haplotypes, so to date it has not been possible to establish whether C4 null alleles are causal in lupus. Recently the development of autoimmunity in patients treated with TNF-alpha antagonists has stimulated interest in the possible role of TNF in SLE [48]–[50]. However, the limited number of polymorphisms genotyped and the strong LD between certain *TNF* alleles and the *B8-DR3* haplotype again restrict interpretation of these data.

In 2002, a family-based association study [51] identified three microsatellite-inferred risk haplotypes in European lupus families: DRB1*1501/DQB1*0602, DRB1*0301/DQB1*0201, and DRB1*0801/DQB1*0402. Further analysis of ancestral recombinants could only delimit the disease-associated region to 1 Mb of the MHC, encompassing class II and class III.

Taking the above into account, it is not surprising that our analysis demonstrates predominant association with variants linked to *DR3-* and *DR2*-bearing ancestral haplotypes in SLE (Figure 1, bottom). The observed DRB1*0401 signal principally arises from Mexican Mestizo and Hispanic cohorts in whom this allele is uncommon (frequency ∼1%). This association has not been well described and warrants further study. Two further class II alleles, HLA-DQA1*0401 and HLA-DQB1*0402, reside on a *DR8* haplotype, which is infrequent in European populations (frequency ∼2%). Our pooled analysis highlights the importance of polymorphism within *HLA-DR3*–containing haplotypes in lupus susceptibility. The remaining association signals largely arise from alleles of the class II genes *HLA-DRB1*, *HLA-DQA1,* and *HLA-DQB1* (see Dataset S3 for further information).

## Inflammatory Bowel Diseases: Ulcerative Colitis and Crohn's Disease

CD and UC are related inflammatory diseases of the gastrointestinal tract commonly known as inflammatory bowel diseases (IBD, MIM 266600). Several independent genome-wide scans in both CD and UC have shown evidence of linkage to the MHC (*IBD3* locus) [52]–[58]. It has been suggested that this region may exert a greater effect in susceptibility to UC rather than CD (with genetic contribution estimates of 60%–100% for UC and 10% for CD) [59].

The most consistent associations in UC are with the class II alleles HLA-DRB1*1502 and HLA-DRB1*0103 [60]–[67]. HLA-DRB1*1502 has shown association to UC in the Japanese population, where it is highly prevalent (20%–25%) [61],[63],[65],[66], but also in European populations, where it is rare (less than 1%) [68]. HLA-DRB1*0103 represents the most reproducible association observed to date in UC [60], [62], [67], [69]–[72]; however, it has a low prevalence of less than 2% in Europeans.

In contrast to UC, four separate class II alleles show reproducible association with CD: HLA-DRB1*07, HLA-DRB1*0103, HLA-DRB1*04, and HLA-DRB3*0301 [67]. HLA-DRB1*07 is the most consistently replicated association between the MHC and CD [67], [73]–[75], and more specifically with ileal disease [73]–[75]. DRB1*0103, also associated with susceptibility to UC [60], [67], [73]–[76], shows subphenotype specificity to colonic CD [73]–[75]. HLA-DRB1*04 has shown a weak but reproducible association [61],[67],[74],[77],[78] to CD, predominantly in patients of Japanese origin. Finally, HLA-DRB3*0301 was also identified in CD [67],[73]; however; this locus has been evaluated in only a few studies.

Our pooled analysis substantiates the association of UC to HLA-DRB1*0103 and HLA-DRB1*1502 (Figure 2, top). We also detect a weak predisposing effect of the microsatellite allele *MICA5.1*, supporting previous non-replicated reports of association with polymorphisms of the MHC class I-related gene, *MICA*, and UC [66],[79],[80]. Other novel associations in UC are demonstrated for *HLA-DR5* (and its subspecificities *HLA-DR11* and *-DR12*), *HLA-A19* and *HLA-A24*.

In CD, we confirm significant association signals arising from alleles/haplotypes related to HLA-DRB1*0103, HLA-DRB1*04, HLA-DR7, and HLA-DRB3*0301 (Figure 2, middle). We also confirm previously reported association signals with *HLA-B18* and *HLA-B21*, and identify novel associations to *HLA-DR6* (encompassing HLA-DRB1*1401), *HLA-DR8* (including HLA-DRB1*0802 and *0803), and *HLA-DR10*. Finally, we substantiate previous reports of association [81]–[85] of the *TNF* promoter polymorphism *TNF-857T* with CD, and further show association with *TNF-1031C* and *TNF-863A* (see Dataset S4 for further information).

## Rheumatoid Arthritis

Rheumatoid arthritis (RA, MIM 180300) is a chronic systemic disorder, the hallmark of which is an inflammatory polyarthritis. It has been estimated that the MHC accounts for approximately one-third of the overall genetic component of RA risk [86],[87].

Much, but probably not all, of the risk attributable to the MHC is associated with variation at *HLA-DRB1*. When the susceptible *HLA-DR* subtypes were considered as a group, Gregersen et al. noted a shared amino acid sequence at positions 70–74 of the HLA-DRB1 protein [88]. These alleles are now known collectively as “shared epitope” alleles because of the related sequence composition in the third hypervariable region: the susceptibility alleles result in missense amino acid changes, where the shared susceptibility amino acid motif is ^70^Q/R-K/R-R-A-A^74^. The most common (>5% population frequency) *HLA-DRB1* shared epitope susceptibility alleles include *0101, *0401, and *0404 in individuals of European ancestry, and *0405 and *0901 in individuals of Asian ancestry. The strength of genetic association to RA susceptibility differs across the *HLA-DRB1* alleles, there being at least two classes of *HLA-DRB1* risk alleles, high and moderate. In general, the DRB1*0401 allele exhibits a high level of risk, with a relative risk (RR) of approximately 3. The DRB1*0101, *0404, *1001, and *0901 alleles exhibit a more moderate RR in the range of 1.5.

Several studies suggest that additional genes within the MHC likely contribute to disease susceptibility once the effect of *HLA-DRB1* has been taken into consideration [89]–[93]. For example, an extended haplotype that includes HLA-DRB1*03 alleles may be associated with RA [89]. The associated haplotype spans ∼500 kb, and contains MHC class III genes, including the *TNF* locus implicated in other studies [92],[94],[95].

Genetic variation at the *HLA-DRB1* gene is clearly associated with RA. Our pooled analysis (Figure 2, bottom) also suggests the existence of high and moderate risk alleles. The *0401 and *0405 alleles have ORs in the range of 3.5, with lower-limit 95% CIs >3.0. The next class of *HLA-DRB1* alleles have ORs in the 1.5–2.0 range. These alleles include: *0101, *0404, *0901, and *1001; additional *DR4* alleles are also included in this group (*Dw14_0404-0408* alleles). Our analysis also supports the hypothesis that genetic variation located a significant genomic distance away from the *HLA-DRB1* gene—and thus possibly not in LD with any of the *HLA-DRB1* risk alleles—also appears associated with RA risk. For example, alleles near the *TNF* locus appear associated with RA (see Dataset S5 for further information).

## Conclusions

Our review and pooled analysis of MHC association in MS, T1D, SLE, CD, UC, and RA corroborate established data and identify predisposing variants that have not been previously appreciated. Apart from IBD, this is the first time, to our knowledge, that the published literature regarding MHC genetics has been systematically analyzed in these diseases. Specifically, we corroborate established data showing that *HLA-DR2* and *-DR3* containing haplotypes harbor lupus and MS susceptibility alleles in European populations. We also demonstrate a putative predisposing effect of *HLA-DR4* haplotypes in these diseases: HLA-DRB1*0401 (or a variant in LD with this allele) in non-European lupus cohorts and several different HLA-DRB1*04 alleles in MS. In RA we emphasize the high risk of both HLA-DRB1*0401 and *0405 despite apparent molecular heterogeneity. We confirm an established association with haplotypes containing *DR3, DR4,* and *DR9* in T1D. Furthermore we find hitherto unidentified class I (*MICA5.1* and *HLA-A19*) and class II (*HLA-DR5, -6, -8,* and *-10*) association signals in IBD.

**Figure 3:**
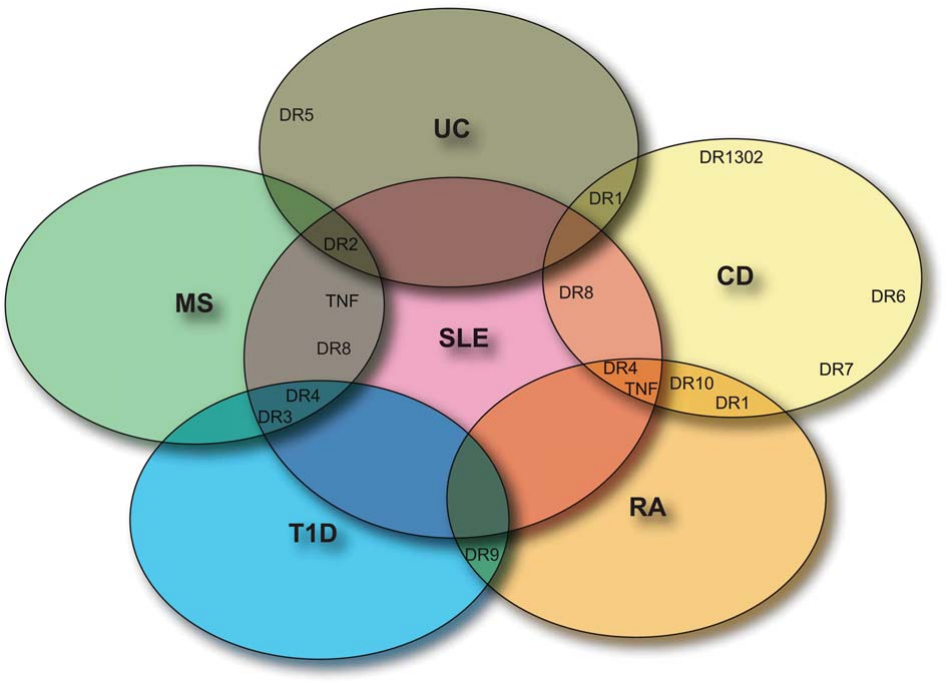
Illustration of the principal shared and distinct MHC haplotype associations in six immune-mediated diseases demonstrated by this pooled analysis. This Venn diagram illustrates the principal shared and distinct MHC haplotype associations in MS, T1D, SLE, US, CD, and RA demonstrated by this pooled analysis. SLE is displayed at the centre of the figure, because it is a multisystem autoimmune disease, while the surrounding diseases are predominantly, though not exclusively, organ-specific. The *HLA-DR* variants indicated in the figure represent their respective extended haplotypes; *TNF-alpha* polymorphisms signify association at this gene alone.

The pooled analysis highlights a number of commonalities as well as differences across the six diseases (Figure 3). The most frequently shared disease susceptibility alleles arise from *HLA-DR4* haplotypes, which are observed in all cases except UC. *HLA-DR3* haplotypes are clearly important in disease predisposition for SLE, MS, and T1D, while *DR9* haplotypes are seen in T1D and RA. CD, UC, and RA share *DR1* haplotypes, although the specific *DR1* alleles differ, as is the case in SLE, MS, and UC, where different *DR2* haplotypes are observed. The various *TNF* polymorphisms that show disease predisposition in RA, CD, MS, and SLE demonstrate an interesting paradox, as therapeutic TNF-alpha blockade for CD and RA is associated with the development of demyelination and antinuclear antibodies. The susceptibility alleles/haplotypes identified in this review therefore suggest both common and disease-specific pathogenetic mechanisms in autoimmunity.

We emphasize that nearly all association studies of the MHC in autoimmune and inflammatory disease to date have been limited to a subset of ∼20 genes and have been performed in small cohorts of predominantly European origin. These genes include the classical HLA loci (*HLA-A, -B, -C, -DRB, -DQA, -DQB, -DPA,* and *-DPB*), *TNF*, *LTA*, *LTB*, the *TAP* genes, *MICA, MICB,* and the complement loci (*C2*, *C4*, *CFB*). Moreover, most of these studies individually investigated only a small proportion of this limited subset of genes, thus impeding our ability to compare the strength of association across studies for this limited number of loci. Given that there are 421 genetic loci currently annotated to the xMHC, approximately 252 (60%) of which are thought to be expressed, it is necessary that a more comprehensive approach to the study of the MHC in disease is undertaken in conjunction with conditional analyses that address the issue of independent susceptibility loci within the region. In order to differentiate the effects of tightly linked loci, a dense map of variation is needed in large cohorts of ethnically (thus haplotypically) diverse populations, so that rare, distinguishing recombination events can be identified. Conditional analyses can then be applied to separate allelic from haplotypic association. Such statistical analyses are only now possible with respect to the MHC. Indeed, the recently published MHC single nucleotide polymorphism studies in MS [19], SLE [96] and T1D [97] and the forthcoming IMAGEN (International Major Histocompatibility Complex and Autoimmunity Genetics Network) consortium data prove the utility of these experiments. Fine-mapping and replication of the resulting independent association signals in appropriately powered European and non-European cohorts will present future challenges prior to moving forward with functional studies. Undoubtedly, the next one to two years will be witness to the identification of the causal variants within the MHC in autoimmune disease.

## Supporting Information

Table S1 Pooled analysis input files for (a) multiple sclerosis, (b) type 1 diabetes, (c) systemic lupus erythematosus, (d) ulcerative colitis, (e) Crohn disease, and (f) rheumatoid arthritis.
(0.44 MB XLS)

Table S2 Complete pooled analysis results for each MHC allele or phenotype for all diseases indicating numbers of cases and controls, number of studies, odds ratio and 95% confidence interval. Variants highlighted in yellow are positively* associated with disease (defined as lower confidence interval >1.0) and are illustrated in Figures 1 and 2.
(0.11 MB XLS)

Table S3 Comparison of serological, T lymphocyte and molecular specificities for MHC variants showing positive* association in pooled analysis (defined as lower confidence interval >1.0).
(0.02 MB XLS)

Dataset S1 Review and pooled analysis of MHC association with multiple sclerosis.
(0.03 MB PDF)

Dataset S2 Review and pooled analysis of MHC association with type 1 diabetes.
(0.08 MB PDF)

Dataset S3 Review and pooled analysis of MHC association with systemic lupus erythematosus.
(0.03 MB PDF)

Dataset S4 Review and pooled analysis of MHC association with inflammatory bowel disease.
(0.03 MB PDF)

Dataset S5 Review and pooled analysis of MHC association with rheumatoid arthritis.
(0.07 MB PDF)

Text S1 Supplementary Materials and Methods.
(0.09 MB PDF)

Text S2 Nomenclature of HLA and Non-HLA Alleles.
(0.09 MB PDF)
