# Supplementary material for: Defining the Role of the MHC in Autoimmunity: A Review and Pooled Analysis
Source: PLoS Genet. 2008 Apr 25;4(4):e1000024. doi: 10.1371/journal.pgen.1000024 (PMC2291482; doi:10.1371/journal.pgen.1000024)
Supplement: Dataset S1 — Review and pooled analysis of MHC association with multiple sclerosis. (0.03 MB PDF) [file pgen.1000024.s004.pdf]

Data S1

Multiple Sclerosis

Multiple sclerosis (MS, MIM 126200) is a chronic inflammatory demyelinating disorder of the central nervous system (reviewed in [1,2]). The disease has a predilection for females and may manifest as relapsing and/or progressive forms. The only region of the genome that has shown consistent evidence of linkage and association with multiple sclerosis (MS) is the HLA locus (reviewed in [3]). The association of the *HLA-DR2* haplotype with multiple sclerosis was first noted in 1972 [4] and remains one of the most reproduced findings in MHC genetics [5-15].

In all of the linkage scans performed to date, the HLA locus is the only one to have achieved genome-wide significance as an MS susceptibility locus both in a meta-analysis of linkage studies [10] and in a subsequent large high-resolution linkage screen in MS [11]. An initial SNP-based association study of the MHC as well as more focused efforts dissecting the effects of the MHC in MS have recently confirmed the preeminent role of the *DRB1*1501* (encompassed within the *DR2* specificity) haplotype in MS in populations of northern European ancestry [12-14]. Indeed, populations with a high concentration of the *DRB1*1501-DQB1*0602* haplotype, such as northern Scotland, exhibit some of the highest known prevalence rates of MS [16].

Due to strong LD within the MHC, it remains unclear whether the primary driver for the association of the HLA locus to MS is the *DQB1*0602* allele or the *DRB1*1501* allele (reviewed in [3]). However, evidence is mounting that, of these two alleles, *DRB1*1501* appears to be the allele with stronger evidence of association both in African-Americans [17] and, possibly, in

European populations (PLD, unpublished data). The long-range LD noted within the *DRB1*1501-DQB1*0602* haplotype and its effect strength (OR = 2.7 for one copy of the DRB*1501 allele, [18]) hinders the interpretation of other associations in this region, even those several megabases away in the MHC class I region. Thus, it is uncertain whether MHC class I associations are truly independent of the *DRB1*1501-DQB1*0602* haplotype effect [3], though some evidence suggests that the *DRB1*1501-DQB1*0602* associated *HLA-A3* allele may be an independent susceptibility signal in MS [19]. Investigators in the Netherlands have also reported an association with the microsatellite *HLA C1_3_2*354* that is independent of *DR2*, although in strong LD with *DR3* [20]. A more recent study of a different family-based cohort failed to demonstrate such independent *HLA-A* or *-B* effects [21]. On the other hand, another study that took both the *DR15* and *DR3* effects into account offers substantial evidence that the *HLA C*05* allele may be independently associated to MS susceptibility [14]. Thus, the role of class I alleles remains open for now and awaits the interrogation of much larger subject samples in an effort to powerfully address the issue of *DRB1*-independent susceptibility loci.

Within the *DRB1* gene itself, there appears to be evidence for allelic heterogeneity, particularly in non-European populations [22]. Nonetheless, the haplotype tagged by *HLA DRB1*1501* remains a risk haplotype for MS in non-European populations, especially when one considers the typical disseminated forms of demyelinating disease captured by the MS diagnostic rubric [23-25]. Barcellos and colleagues [13] have recently explored the question of allelic heterogeneity in 1339 families of European ancestry with

MS, and this robust analysis reports a dominant effect for *DRB1*15*, a recessive effect for *DRB1*03*, a protective effect for *DRB1*14*, as well as evidence for interaction between *DRB1*15* and *DRB1*08* in increasing susceptibility to MS. A separate study that shared some subjects with the latter study suggests that the *HLA DRB1*0103* may also be a susceptibility allele, once the effects of *DRB1*15* and *DRB1*03* are taken into account [14]. A small study of Portuguese subjects recently also found an association to *HLA DRB1*03* [26], as did earlier studies of Sardinian families [27]. Mediterranean populations such as the Sardinians also display associations to *DR4* (reviewed in [1], and the *DR4* effect has now been demonstrated in another Italian population consisting of families with MS and other autoimmune diseases [28]. Finally, as with the *DRB1*14* allele described above, several HLA class II alleles (*DR1, DR7,* and *DR11*) have been implicated in protection against MS [29], while *HLA-A*0201* has been shown to reduce *DRB1*15*-associated risk [19]. Such protective effects are notoriously difficult to distinguish from under representation of certain alleles in the face of the effects of *DRB1*15*, and therefore these findings await further validation.

Evidence for association of other *DRB1* alleles comes from other human populations. In Japan, disseminated MS similar to that seen in Europeans may be associated with *DRB1*1501*, but the association of the *DRB1*1501* haplotype with the opticospinal MS variant fails to reach significance (reviewed in [24]). Instead, opticospinal MS may be associated with *DPB1*0501* and *DPB1*0301* in the Japanese population [24,30]. An

association with *DR6* in Japanese has also been reported, but without replication [31]. In the Turkish population, an association has been suggested with *DR4* ([32]reviewed in [33]). The same haplotype is also reported to be associated with susceptibility to MS in Mexican Mestizos [34]. In African-Americans, MS association has also been demonstrated with *DRB1*0301* and *DRB1*1503* in addition to the expected *DRB1*1501* [17].

Outside of MHC genes, polymorphisms in the immunologically relevant class III genes, *TNF* and *NOTCH4*, have been associated with MS, but this association was shown to be secondary to that of *DRB1*1501* [35]. At least 10 small studies have shown no HLA-independent association with microsatellites and SNPs in linkage disequilibrium with the *TNF* gene (ibid.), though one as yet unreplicated larger study [36] suggests an association in a Spanish population. Recently, a small study of Tasmanian families has highlighted a haplotype containing a myelin oligodendrocyte glycoprotein (MOG) allele after conditioning for the effect of *HLA-DRB1*15* [37]. Thus, the role of non-HLA genes also remains an area of active investigation that will benefit from much larger studies that comprehensively interrogate genetic variation within the extended MHC in MS.

As expected, our pooled analysis of studies exploring the role of the MHC with MS susceptibility highlights the pre-eminent role of the extended haplotype defined by *HLA DRB1*1501* and *DQB1*0602* (Figure 1). Whether typed as *Dw2, DR2, DR15*, or *DRB1*1501*, the presence of a *1501* allele of the *HLA-DRB1* gene is associated with an OR > 2.0 for susceptibility to MS. This allele,

or another allele in strong LD with it, is therefore an important risk factor for the onset of this disease. Given the extended LD that haplotypes bearing *HLA-DRB1*1501* display, the problem of identifying the causal variant or variants within the haplotype is extremely challenging. In the data available to us, we have evaluated the alleles *HLA-A3, -B7, -DQA1*0102, -DQB1*0602* and *TNFa11* which lie on the ancestral *DRB1*1501*-containing haplotype. For the most part, the pooled analysis suggests that none of these alleles is as strongly associated to MS susceptibility as is *HLA-DRB1*1501*; thus, in general, the more modest effects of these other alleles are probably due to LD with *HLA-DRB1*1501*. This result is consistent with the exclusion of *DQB1*0602*, the allele in highest LD with *HLA DRB1*1501*, as a risk allele in African-Americans with MS [17].

Two other ancestral haplotypes, *DR3* and *DR4*, appear to play a role in MS susceptibility, although the effect of these haplotypes on disease is more modest than that of the *DR2* haplotypes. Figure 1 displays those alleles found on *DR3* haplotypes that show significant association with susceptibility to MS in our pooled analysis. Here, it is less clear than in the *HLA DRB1*1501* analysis whether the *DRB1*0301* allele is primarily driving the association or whether one of the alleles of *TNF*, for example, could be in stronger LD with a risk allele. On the other hand, the *DR4* haplotypes seem to display their strongest association with their *HLA-DRB1* alleles: four different *DR4* alleles – *0402, 0403, 0404,* and *0405* – display significant association in our analysis. The *DR4* haplotype-associated *DQB1*0302* allele is also associated with MS susceptibility but appears to have a more modest effect than the *DRB1* alleles, and therefore, as with the *DR2* haplotypes, alleles within the *DRB1*

gene may be playing a causal role. However, given the relatively rare frequencies of these alleles, one must interpret our results cautiously: large-scale studies will be needed to more accurately evaluate the effect of the various *DR4* haplotypes. Nonetheless, our analysis suggests that, while the population risk of *DR4* haplotypes on MS susceptibility may be relatively small, those rare individuals bearing these alleles may have a large increase in disease risk; the odds ratios for *DR4* haplotypes range from 1.7 for *DRB1*0404* to 2.8 for *DRB1*0403*.

In MS, our pooled analysis highlights the class II gene, *HLA-DRB1* as the primary candidate for MS susceptibility at the MHC, with a number of different alleles contributing to disease risk. Evidence implicating the *HLA-DR* specificities, *DR2* and *DR4,* includes the genetic analyses summarized here and also the results of functional studies. While the key target antigens in MS remain elusive, human and animal studies have shown that different protein components of myelin, including myelin basic protein (MBP), myelin oligodendrocyte glycoprotein (MOG), and myelin proteolipid protein (PLP), all contain peptide sequences that can be presented by DR2 or DR4. *DR2* alleles have been studied extensively, with reactivity to MBP 84-102 [38,39]; PLP 95-116, 184-199 and 190-206 [40,41]; and overlapping MOG peptides [42]. The data on *DR4* alleles is limited, with the MBP 111-129 epitope being recognized in subjects with *DRB1*0401* [43], but PLP and MOG epitopes were also recognized in one study where subjects with at least one *DR4* allele were studied [44]. Further studies are required to fully elucidate and validate the allelic heterogeneity seen at *DRB1*, particularly in terms of the role of rare alleles such as *DRB1*0403*; recent efforts are offering increasing evidence

supporting the role of the more common *DRB1*03* allele in disease susceptibility [13,14].

REFERENCES

1. Compston A, Coles A (2002) Multiple sclerosis. Lancet 359: 1221-1231.
2. Hauser SL, Oksenberg JR (2006) The neurobiology of multiple sclerosis: genes, inflammation, and neurodegeneration. Neuron 52: 61-76.
3. Harbo HF, Lie BA, Sawcer S, Celius EG, Dai KZ, et al. (2004) Genes in the HLA class I region may contribute to the HLA class II-associated genetic susceptibility to multiple sclerosis. Tissue Antigens 63: 237-247.
4. Jersild C, Svejgaard A, Fog T (1972) HL-A antigens and multiple sclerosis. Lancet 1: 1240-1241.
5. Winchester R, Ebers G, Fu SM, Espinosa L, Zabriskie J, et al. (1975) B-cell alloantigen Ag 7a in multiple sclerosis. Lancet 2: 814.
6. Compston DA, Batchelor JR, McDonald WI (1976) B-lymphocyte alloantigens associated with multiple sclerosis. Lancet 2: 1261-1265.
7. Olerup O, Hillert J (1991) HLA class II-associated genetic susceptibility in multiple sclerosis: a critical evaluation. Tissue Antigens 38: 1-15.
8. Stewart GJ, Teutsch SM, Castle M, Heard RN, Bennetts BH (1997) HLA-DR, -DQA1 and -DQB1 associations in Australian multiple sclerosis patients. Eur J Immunogenet 24: 81-92.
9. Haines JL, Terwedow HA, Burgess K, Pericak-Vance MA, Rimmler JB, et al. (1998) Linkage of the MHC to familial multiple sclerosis suggests genetic heterogeneity. The Multiple Sclerosis Genetics Group. Hum Mol Genet 7: 1229-1234.
10. GAMES (2003) A meta-analysis of whole genome linkage screens in multiple sclerosis. J Neuroimmunol 143: 39-46.
11. Sawcer S, Ban M, Maranian M, Yeo TW, Compston A, et al. (2005) A high-density screen for linkage in multiple sclerosis. Am J Hum Genet 77: 454-467.
12. Lincoln MR, Montpetit A, Cader MZ, Saarela J, Dyment DA, et al. (2005) A predominant role for the HLA class II region in the association of the MHC region with multiple sclerosis. Nat Genet 37: 1108-1112.
13. Barcellos LF, Sawcer S, Ramsay PP, Baranzini SE, Thomson G, et al. (2006) Heterogeneity at the HLA-DRB1 locus and risk for multiple sclerosis. Hum Mol Genet 15: 2813-2824.
14. Yeo TW, De Jager PL, Gregory SG, Barcellos LF, Walton A, et al. (2007) A second major histocompatibility complex susceptibility locus for multiple sclerosis. Ann Neurol 61: 228-236.
15. Hafler DA, Compston A, Sawcer S, Lander ES, Daly MJ, et al. (2007) Risk alleles for multiple sclerosis identified by a genomewide study. N Engl J Med 357: 851-862.
16. Downie AW, Phadke JG (1984) The chief scientist reports ... multiple sclerosis in North East Scotland. Health Bull (Edinb) 42: 151-156.

17. Oksenberg JR, Barcellos LF, Cree BA, Baranzini SE, Bugawan TL, et al. (2004) Mapping multiple sclerosis susceptibility to the HLA-DR locus in African Americans. Am J Hum Genet 74: 160-167.
18. Barcellos LF, Oksenberg JR, Begovich AB, Martin ER, Schmidt S, et al. (2003) HLA-DR2 dose effect on susceptibility to multiple sclerosis and influence on disease course. Am J Hum Genet 72: 710-716.
19. Fogdell-Hahn A, Ligers A, Gronning M, Hillert J, Olerup O (2000) Multiple sclerosis: a modifying influence of HLA class I genes in an HLA class II associated autoimmune disease. Tissue Antigens 55: 140-148.
20. de Jong BA, Huizinga TW, Zanelli E, Giphart MJ, Bollen EL, et al. (2002) Evidence for additional genetic risk indicators of relapse-onset MS within the HLA region. Neurology 59: 549-555.
21. Chao MJ, Barnardo MC, Lui GZ, Lincoln MR, Ramagopalan SV, et al. (2007) Transmission of class I/II multi-locus MHC haplotypes and multiple sclerosis susceptibility: accounting for linkage disequilibrium. Hum Mol Genet 16: 1951-1958.
22. Sawcer S, Compston A (2006) Multiple sclerosis: light at the end of the tunnel. Eur J Hum Genet 14: 257-258.
23. Noseworthy JH, Lucchinetti C, Rodriguez M, Weinshenker BG (2000) Multiple sclerosis. N Engl J Med 343: 938-952.
24. Kira J (2003) Multiple sclerosis in the Japanese population. Lancet Neurol 2: 117-127.
25. Brassat D, Salemi G, Barcellos LF, McNeill G, Proia P, et al. (2005) The HLA locus and multiple sclerosis in Sicily. Neurology 64: 361-363.
26. Silva AM, Pereira C, Bettencourt A, Carvalho C, Couto AR, et al. (2007) The role of HLA-DRB1 alleles on susceptibility and outcome of a Portuguese Multiple Sclerosis population. J Neurol Sci 258: 69-74.
27. Marrosu MG, Murru R, Murru MR, Costa G, Zavattari P, et al. (2001) Dissection of the HLA association with multiple sclerosis in the founder isolated population of Sardinia. Hum Mol Genet 10: 2907-2916.
28. Laroni A, Calabrese M, Perini P, Albergoni MP, Ranzato F, et al. (2006) Multiple sclerosis and autoimmune diseases: epidemiology and HLA-DR association in North-east Italy. J Neurol 253: 636-639.
29. Giordano M, D'Alfonso S, Momigliano-Richiardi P (2002) Genetics of multiple sclerosis: linkage and association studies. Am J Pharmacogenomics 2: 37-58.
30. Fukazawa T, Yamasaki K, Ito H, Kikuchi S, Minohara M, et al. (2000) Both the HLA-CPB1 and -DRB1 alleles correlate with risk for multiple sclerosis in Japanese: clinical phenotypes and gender as important factors. Tissue Antigens 55: 199-205.
31. Naito S, Kuroiwa Y, Itoyama T, Tsubaki T, Horikawa A, et al. (1978) HLA and Japanese MS. Tissue Antigens 12: 19-24.
32. Saruhan-Direskeneli G, Esin S, Baykan-Kurt B, Ornek I, Vaughan R, et al. (1997) HLA-DR and -DQ associations with multiple sclerosis in Turkey. Hum Immunol 55: 59-65.
33. Compston A (1999) The genetic epidemiology of multiple sclerosis. Philos Trans R Soc Lond B Biol Sci 354: 1623-1634.
34. Alaez C, Corona T, Ruano L, Flores H, Loyola M, et al. (2005) Mediterranean and Amerindian MHC class II alleles are associated with multiple sclerosis in Mexicans. Acta Neurol Scand 112: 317-322.

35. Duvefelt K, Anderson M, Fogdell-Hahn A, Hillert J (2004) A NOTCH4 association with multiple sclerosis is secondary to HLA-DR*1501. Tissue Antigens 63: 13-20.
36. Fernandez-Arquero M, Arroyo R, Rubio A, Martin C, Vigil P, et al. (1999) Primary association of a TNF gene polymorphism with susceptibility to multiple sclerosis. Neurology 53: 1361-1363.
37. Rubio JP, Bahlo M, Stankovich J, Burfoot RK, Johnson LJ, et al. (2007) Analysis of extended HLA haplotypes in multiple sclerosis and narcolepsy families confirms a predisposing effect for the class I region in Tasmanian MS patients. Immunogenetics 59: 177-186.
38. Ota K, Matsui M, Milford EL, Mackin GA, Weiner HL, et al. (1990) T-cell recognition of an immunodominant myelin basic protein epitope in multiple sclerosis. Nature 346: 183-187.
39. Jingwu Z, Medaer R, Hashim GA, Chin Y, van den Berg-Loonen E, et al. (1992) Myelin basic protein-specific T lymphocytes in multiple sclerosis and controls: precursor frequency, fine specificity, and cytotoxicity. Ann Neurol 32: 330-338.
40. Ohashi T, Yamamura T, Inobe J, Kondo T, Kunishita T, et al. (1995) Analysis of proteolipid protein (PLP)-specific T cells in multiple sclerosis: identification of PLP 95-116 as an HLA-DR2,w15-associated determinant. Int Immunol 7: 1771-1778.
41. Greer JM, Dyer CA, Pakaski M, Symonowicz C, Lees MB (1996) Orientation of myelin proteolipid protein in the oligodendrocyte cell membrane. Neurochem Res 21: 431-440.
42. Wallstrom E, Khademi M, Andersson M, Weissert R, Linington C, et al. (1998) Increased reactivity to myelin oligodendrocyte glycoprotein peptides and epitope mapping in HLA DR2(15)+ multiple sclerosis. Eur J Immunol 28: 3329-3335.
43. Muraro PA, Vergelli M, Kalbus M, Banks DE, Nagle JW, et al. (1997) Immunodominance of a low-affinity major histocompatibility complex-binding myelin basic protein epitope (residues 111-129) in HLA-DR4 (B1*0401) subjects is associated with a restricted T cell receptor repertoire. J Clin Invest 100: 339-349.
44. Yentur SP, Akman-Demir G, Eraksoy M, Saruhan-Direskeneli G (2002) Autoreactivity to myelin antigens related to HLA associations with multiple sclerosis. Mult Scler 8: 278-283.
