# Supplementary material for: Defining the Role of the MHC in Autoimmunity: A Review and Pooled Analysis
Source: PLoS Genet. 2008 Apr 25;4(4):e1000024. doi: 10.1371/journal.pgen.1000024 (PMC2291482; doi:10.1371/journal.pgen.1000024)
Supplement: Dataset S2 — Review and pooled analysis of MHC association with type 1 diabetes. (0.08 MB PDF) [file pgen.1000024.s005.pdf]

Data S2

Type 1 Diabetes

Type 1 diabetes (T1D, MIM 222100) is a chronic autoimmune disease characterized by T cell-mediated destruction of pancreatic islet beta cells resulting in irreversible insulin deficiency and long-term dysfunction of several organs and tissues. Five main auto-antibodies, directed against islet antigens, are associated with disease: antibodies against insulin (IAA), glutamic acid decarboxylase-65 (GAD65), islet cell antigens (ICA), protein tyrosine phosphatase, ICA512 or IA2 (IA2) and zinc transporter ZnT8 (ZnT8A) [1]. The incidence if T1D varies greatly between different populations, ranging from 0.1/100 000/year in China to 36/100 000/year in Sardinia and Finland [2].

There is no doubt that the major genetic contribution to T1D susceptibility arises from the MHC [3]. This region, also designated *IDDM1*, is the only locus linked with T1D in every genome-wide screen to date. Several other genomic intervals have been linked to disease susceptibility, most notably the *IDDM2* locus on chromosome 11 in the region of the insulin gene [4]. Approximately 50% of the total genetic contribution to T1D is attributable to the MHC [5] in comparison to only 15% in multiple sclerosis [6].

T1D susceptibility conferred by *IDDM1* is complex and represents the combined effects of several susceptibility genes within the MHC [3]. Initial case-control studies of the MHC in T1D demonstrated associations with *HLA-B* serotypes [7]. These were subsequently shown to be due to linkage disequilibrium (LD) with the class II loci, *HLA-DR* and *HLA-DQ* [8]. It is now evident that the most important genes involved in T1D susceptibility at *IDDM1*

are *HLA-DRB1*, *-DQA1*, and *-DQB1*. To date most evidence supports a role for *HLA-DQ* as the major disease predisposing locus [9,10].

Most genetic studies in T1D have been undertaken in white Caucasian populations and consistently demonstrate disease predisposition with the haplotypes, *DRB1*04-DQA1*0301-DQB1*0302* and *DRB1*03-DQA1*0501-DQB1*0201* [8]. Heterozygosity for this combination of alleles confers the highest risk for T1D in several populations in a synergistic manner [8]. The formation of specific *trans* DQ dimers by transcomplementation between *DQA1* and *DQB1* alleles on homologous chromosomes (*DQA1*0301/DQB1*0201* and *DQA1*0501/DQB1*0302*) may be responsible for the increase in heterozygote risk [3,11,12].

More than 90% of Caucasian individuals with T1D carry at least one of the two risk haplotypes: *DRB1*04-DQA1*0301-DQB1*0302* and *DRB1*03-DQA1*0501-DQB1*0201* compared with around 40% of the general population [13]. Therefore roughly 10% will carry neither haplotype. *DR3/DR4* heterozygosity appears to influence age of onset in TID. This genotype occurs with greatest frequency in children who develop T1D before age 5 (50%) and least frequently in adults presenting with disease (20-40%), compared with a US population prevalence of 2.4% [14]. In addition, *DR3/DR4* heterozygotes possess a 5% risk of developing T1D by age 15 [15].

DR4 (*DRB1*0405-DQB1*0401*) and *DR9* (*DRB1*0901-DQB1*0303*) have shown association with T1D in Japanese and Korean populations [16]. The low frequency of the disease-associated *DR3* and *DR4* haplotypes may contribute to the reduced incidence of T1D in these non-white populations [8,17,18].

The nature of the *HLA-DR* association in T1D remains unclear. LD with *HLA-DQ* alleles may account for part of this association as *DRB1*03* is in LD with *DQA1*0501-DQB1*0201* and *DRB1*04* is in LD with *DQA1*0301-DQB1*0302* [3]. Other *DRB1* alleles, in particular *DRB1*04*, may also modify the risk present at the *DQ* locus. *DRB1*0401* and *DRB1*0405* have been associated with increased disease risk in several populations independent of *DQA1*0301-DQB1*0302*, while *DRB1*0403* and *DRB1*0406* appear to confer protection from disease. The protective effect of *DRB1*0403* appears to be dominant in that this allele was shown to overcome disease susceptibility in individuals carrying the highest risk genotype *DQA1*0301-DQB1*0302* and *DQA1*0501-DQB1*0201* [8]. The effect of predisposing *DR3*- and *DR4*-containing haplotypes is more consistent with a recessive model of inheritance [3].

The *DQA1*0102-DQB1*0602* haplotype confers strong protection from T1D in Caucasian and Japanese populations [19-21]. Such protection dominates over the susceptibility encoded by the high-risk *DQ* alleles, but is not absolute. In contrast the *DRB1*1501-DQA1*0102-DQB1*0602* haplotype is associated with an increased risk of other autoimmune diseases, such as multiple sclerosis and systemic lupus erythematosus. Different class II alleles, including *DRB1*13-DQB1*0301*, *DRB1*11-DQB1*0301*, *DRB1*01-DQB1*0501*, have shown evidence of protection in other populations [22]. Hence, determination of *HLA-DQ* (and *-DR*) status may prove beneficial in risk stratification for T1D in at-risk autoantibody positive individuals [23].

The presence or absence of various amino acid residues of the DRβ, DQα and DQβ peptide chains may be important in disease susceptibility by altering

the nature of the peptide binding groove. The absence of an aspartic acid residue at position 57 (Asp57) of the DQβ chain and the presence of an arginine residue at position 52 (Arg52) of the DQα peptide have been associated with susceptibility to disease. This hypothesis does not hold true for all T1D susceptibility alleles and the exact contribution of single or multiple amino acid residues to susceptibility remains to be determined [24]. A variety of studies have demonstrated association with HLA alleles and auto-antibody status in T1D; the most consistent findings are those of IAA, ICA, IA2 with *DQ8* (*DQB1*0302*) [25,26] and GAD65 with *DQ2* (*DQB1*0201*) [26-28]. It has yet to be established whether these antibodies are pathogenic or merely occur as a consequence of islet cell destruction.

More recently evidence is accumulating for the role of non-HLA loci within the MHC in susceptibility to T1D. A number of groups have shown that genes, including a polymorphism in *ITPR3* (inositol triphosphate receptor 3), telomeric of class I may contribute to disease predisposition [29-32]. Polymorphisms of the MHC class I polypeptide-related sequence A, *MICA*, in the class III region, may lack an independent effect on genetic risk in T1D given that studies in different populations show inconsistent association of *MICA* alleles with disease and that associated alleles are often in LD with MHC class II risk haplotypes [33,34]. Promoter polymorphisms of another class III gene, *TNF* (tumour necrosis factor alpha), have been extensively studied in T1D [35-48]. Thus far, these associations have also been demonstrated to be secondary to LD with HLA class II alleles. Furthermore, *DR-DQ* independent effects in T1D have been shown with respect to age of

onset for *HLA-DPB1* [49,50], class I genes [51,52] and microsatellites within the class III region [53].

Overall, studies to date suggest that both *DR* and *DQ* genes are important in determining disease risk, but the effects of individual alleles may be modified by the haplotypes on which they are carried [8]. There appears to be a hierarchy of risk alleles from the strongly protective *DQB1*0602* to the highly predisposing *DQB1*0302*. Such a spectrum of risk is also borne out by TDT analysis showing that each HLA-DR/HLA-DQ haplotype has its own individual disease risk which may result from transcomplementation and other haplotypic effects.

The main T1D association signals (29 out of 33) determined by this pooled analysis arise from *DR3*, *DR4* and *DR9*-containing haplotypes and concur with the published literature (Figure 1). The alleles of the *HLA-DR3* haplotypes that show positive association are: *A1, B8, MICA5.1, BfS1, C4A*Q0, DQB1*0201* on AH8.1, *B18, BfF1, DQB1*0201* on AH18.2 and *A33* on the less common AH58.1. Although *DRB1*0301* and *BfF1* show the highest odds ratios (OR) in T1D, the wide confidence intervals suggest these results should be interpreted with caution. Of the four class III-associated alleles that reside on *DR3* haplotypes, *MICA5.1, BfS1, C4A*Q0* are linked with AH8.1 and display OR between 1.4 and 2.8, less than the AH18.2 associated *BfF1* (OR 5.6). These data seem to corroborate previous reports of increased disease susceptibility conferred by AH18.2 compared with AH8.1 [32]. It is of interest to note that *DR3* (OR 3.8) and *DRB1*0301* (OR 6.9) show a greater effect in comparison to *DQB1*0201* (OR 2.9), even though these

two alleles are in strong LD. The opposite is seen with the *DR4* haplotypic association, in relation to *-DR* and *-DQ,* where *DQB1*0302* has an OR of 4.8 and *DR4, DRB1*0401* and *DRB1*0405* exhibit OR between 2 and 3.1. In keeping with the published literature the *HLA-DR9* and *HLA-DRB1*0901* associations observed in our pooled analysis arise from non-European cohorts only. The positively associated *HLA-C* alleles *Cw1, Cw3* and *Cw5*, are all in LD with a range of ancestral haplotypes including the disease predisposing haplotypes, *DR3* and *DR4*. The complement *C4* allele, *C4B5* maps to haplotypes containing *DRB1*0405* and *DRB1*1401*, while *DQB1*03032* may reside on *DRB1*0701* and **0901* haplotypes. DRw53 or DR53 is the antigen encoded by one of the *HLA-DRB* genes, *HLA-DRB4* and is found on *DR4, -7* and *-9* haplotypes.

*DPB1*0201* maps to several disease-associated (*DRB1*0401, 0405, 0301*) and unrelated (*01, 1601, 0701*) haplotypes [54]. Studies demonstrate that *HLA-DPB1* polymorphisms may alter the genetic effects of T1D-associated haplotypes, however, some of these specific *HLA-DPB1* containing haplotypes are low frequency and their effect is small in Caucasian populations [54]. More recently, however, *HLA-DPB1*0402* has been found to significantly protect against the development of anti-islet cell auto-antibodies in a high risk *DR3-DQB1*0201/DR4-DQB1*0302* population [55]. Of the remaining associated alleles *A9* (containing the splits *A23* and the *DR4*-associated *A24*), *B21, B41* and *DMB*0104* do not map to specific ancestral haplotypes and may represent separate signals in T1D.

REFERENCES

1. Wenzlau JM, Juhl K, Yu L, Moua O, Sarkar SA, et al. (2007) The cation efflux transporter ZnT8 (Slc30A8) is a major autoantigen in human type 1 diabetes. Proc Natl Acad Sci U S A 104: 17040-17045.
2. Karvonen M, Viik-Kajander M, Moltchanova E, Libman I, LaPorte R, et al. (2000) Incidence of childhood type 1 diabetes worldwide. Diabetes Mondiale (DiaMond) Project Group. Diabetes Care 23: 1516-1526.
3. Todd JA (1990) Genetic control of autoimmunity in type 1 diabetes. Immunol Today 11: 122-129.
4. Concannon P, Erlich HA, Julier C, Morahan G, Nerup J, et al. (2005) Type 1 diabetes: evidence for susceptibility loci from four genome-wide linkage scans in 1,435 multiplex families. Diabetes 54: 2995-3001.
5. Davies JL, Kawaguchi Y, Bennett ST, Copeman JB, Cordell HJ, et al. (1994) A genome-wide search for human type 1 diabetes susceptibility genes. Nature 371: 130-136.
6. Charron D (2005) Immunogenetics today: HLA, MHC and much more. Curr Opin Immunol 17: 493-497.
7. Nerup J, Platz P, Andersen OO, Christy M, Lyngsoe J, et al. (1974) HL-A antigens and diabetes mellitus. Lancet 2: 864-866.
8. She JX (1996) Susceptibility to type I diabetes: HLA-DQ and DR revisited. Immunol Today 17: 323-329.
9. Thorsby E, Ronningen KS (1993) Particular HLA-DQ molecules play a dominant role in determining susceptibility or resistance to type 1 (insulin-dependent) diabetes mellitus. Diabetologia 36: 371-377.
10. Onengut-Gumuscu S, Concannon P (2002) Mapping genes for autoimmunity in humans: type 1 diabetes as a model. Immunol Rev 190: 182-194.
11. Dorman JS, Bunker CH (2000) HLA-DQ locus of the human leukocyte antigen complex and type 1 diabetes mellitus: a HuGE review. Epidemiol Rev 22: 218-227.
12. Khalil I, Deschamps I, Lepage V, al-Daccak R, Degos L, et al. (1992) Dose effect of cis- and trans-encoded HLA-DQ alpha beta heterodimers in IDDM susceptibility. Diabetes 41: 378-384.
13. Svejgaard A, Platz P, Ryder LP (1983) HLA and disease 1982--a survey. Immunol Rev 70: 193-218.
14. Emery LM, Babu S, Bugawan TL, Norris JM, Erlich HA, et al. (2005) Newborn HLA-DR,DQ genotype screening: age- and ethnicity-specific type 1 diabetes risk estimates. Pediatr Diabetes 6: 136-144.
15. Lambert AP, Gillespie KM, Thomson G, Cordell HJ, Todd JA, et al. (2004) Absolute risk of childhood-onset type 1 diabetes defined by human leukocyte antigen class II genotype: a population-based study in the United Kingdom. J Clin Endocrinol Metab 89: 4037-4043.
16. Kawabata Y, Ikegami H, Kawaguchi Y, Fujisawa T, Shintani M, et al. (2002) Asian-specific HLA haplotypes reveal heterogeneity of the contribution of HLA-DR and -DQ haplotypes to susceptibility to type 1 diabetes. Diabetes 51: 545-551.
17. Ikegami H, Kawaguchi Y, Yamato E, Kuwata S, Tokunaga K, et al. (1992) Analysis by the polymerase chain reaction of histocompatibility

leucocyte antigen-DR9-linked susceptibility to insulin-dependent diabetes mellitus. J Clin Endocrinol Metab 75: 1381-1385.
18. Dorman JS, LaPorte RE, Stone RA, Trucco M (1990) Worldwide differences in the incidence of type I diabetes are associated with amino acid variation at position 57 of the HLA-DQ beta chain. Proc Natl Acad Sci U S A 87: 7370-7374.
19. Greenbaum CJ, Schatz DA, Cuthbertson D, Zeidler A, Eisenbarth GS, et al. (2000) Islet cell antibody-positive relatives with human leukocyte antigen DQA1*0102, DQB1*0602: identification by the Diabetes Prevention Trial-type 1. J Clin Endocrinol Metab 85: 1255-1260.
20. Ikegami H, Fujisawa T, Kawabata Y, Noso S, Ogihara T (2006) Genetics of type 1 diabetes: similarities and differences between Asian and Caucasian populations. Ann N Y Acad Sci 1079: 51-59.
21. Redondo MJ, Kawasaki E, Mulgrew CL, Noble JA, Erlich HA, et al. (2000) DR- and DQ-associated protection from type 1A diabetes: comparison of DRB1*1401 and DQA1*0102-DQB1*0602*. J Clin Endocrinol Metab 85: 3793-3797.
22. Volpini WM, Testa GV, Marques SB, Alves LI, Silva ME, et al. (2001) Family-based association of HLA class II alleles and haplotypes with type I diabetes in Brazilians reveals some characteristics of a highly diversified population. Hum Immunol 62: 1226-1233.
23. Ghodke Y, Joshi K, Chopra A, Patwardhan B (2005) HLA and disease. Eur J Epidemiol 20: 475-488.
24. Gorodezky C, Alaez C, Murguia A, Rodriguez A, Balladares S, et al. (2006) HLA and autoimmune diseases: Type 1 diabetes (T1D) as an example. Autoimmun Rev 5: 187-194.
25. Sabbah E, Savola K, Kulmala P, Veijola R, Vahasalo P, et al. (1999) Diabetes-associated autoantibodies in relation to clinical characteristics and natural course in children with newly diagnosed type 1 diabetes. The Childhood Diabetes In Finland Study Group. J Clin Endocrinol Metab 84: 1534-1539.
26. Graham J, Hagopian WA, Kockum I, Li LS, Sanjeevi CB, et al. (2002) Genetic effects on age-dependent onset and islet cell autoantibody markers in type 1 diabetes. Diabetes 51: 1346-1355.
27. Vandewalle CL, Falorni A, Lernmark A, Goubert P, Dorchy H, et al. (1997) Associations of GAD65- and IA-2- autoantibodies with genetic risk markers in new-onset IDDM patients and their siblings. The Belgian Diabetes Registry. Diabetes Care 20: 1547-1552.
28. Sanjeevi CB, Falorni A, Kockum I, Hagopian WA, Lernmark A (1996) HLA and glutamic acid decarboxylase in human insulin-dependent diabetes mellitus. Diabet Med 13: 209-217.
29. Roach JC, Deutsch K, Li S, Siegel AF, Bekris LM, et al. (2006) Genetic mapping at 3-kilobase resolution reveals inositol 1,4,5-triphosphate receptor 3 as a risk factor for type 1 diabetes in Sweden. Am J Hum Genet 79: 614-627.
30. Aly TA, Ide A, Jahromi MM, Barker JM, Fernando MS, et al. (2006) Extreme genetic risk for type 1A diabetes. Proc Natl Acad Sci U S A 103: 14074-14079.

31. Lie BA, Sollid LM, Ascher H, Ek J, Akselsen HE, et al. (1999) A gene telomeric of the HLA class I region is involved in predisposition to both type 1 diabetes and coeliac disease. Tissue Antigens 54: 162-168.
32. Johansson S, Lie BA, Todd JA, Pociot F, Nerup J, et al. (2003) Evidence of at least two type 1 diabetes susceptibility genes in the HLA complex distinct from HLA-DQB1, -DQA1 and -DRB1. Genes Immun 4: 46-53.
33. Alizadeh BZ, Eerligh P, van der Slik AR, Shastry A, Zhernakova A, et al. (2007) MICA marks additional risk factors for Type 1 diabetes on extended HLA haplotypes: an association and meta-analysis. Mol Immunol 44: 2806-2812.
34. Gambelunghe G, Ghaderi M, Cosentino A, Falorni A, Brunetti P, et al. (2000) Association of MHC Class I chain-related A (MIC-A) gene polymorphism with Type I diabetes. Diabetologia 43: 507-514.
35. Kumar R, Goswami R, Agarwal S, Israni N, Singh SK, et al. (2007) Association and interaction of the TNF-alpha gene with other pro- and anti-inflammatory cytokine genes and HLA genes in patients with type 1 diabetes from North India. Tissue Antigens 69: 557-567.
36. Noble JA, Valdes AM, Lane JA, Green AE, Erlich HA (2006) Linkage disequilibrium with predisposing DR3 haplotypes accounts for apparent effects of tumor necrosis factor and lymphotoxin-alpha polymorphisms on type 1 diabetes susceptibility. Hum Immunol 67: 999-1004.
37. Rassi DM, Wastowski IJ, Simoes RT, Rodrigues S, Deghaide NN, et al. (2006) TNFa-e microsatellite, HLA-DRB1 and -DQB1 alleles and haplotypes in Brazilian patients presenting recently diagnosed type 1 diabetes mellitus. Ann N Y Acad Sci 1079: 300-304.
38. Das SN, Baniasadi V, Kapuria V (2006) Association of -308 TNF-alpha promoter polymorphism with type 1 diabetes in North Indians. Int J Immunogenet 33: 411-416.
39. Deja G, Jarosz-Chobot P, Polanska J, Siekiera U, Malecka-Tendera E (2006) Is the association between TNF-alpha-308 A allele and DMT1 independent of HLA-DRB1, DQB1 alleles? Mediators Inflamm 2006: 19724.
40. Torn C, Hillman M, Sanjeevi CB, Landin-Olsson M (2006) Polymorphisms of TNF microsatellite marker a and HLA-DR-DQ in diabetes mellitus-a study in 609 Swedish subjects. Hum Immunol 67: 527-534.
41. Shbaklo H, Azar ST, Terwedow H, Halaby G, Naja RP, et al. (2003) No association between the -1031 polymorphism in the TNF-alpha promoter region and type 1 diabetes. Hum Immunol 64: 633-638.
42. Bouqbis L, Akhayat O, Garchon HJ, Calafell F, Izaabel H (2003) TNFA-TNFB haplotypes modify susceptibility to type I diabetes mellitus independently of HLA class II in a Moroccan population. Tissue Antigens 61: 72-79.
43. Nishimura M, Obayashi H, Mizuta I, Hara H, Adachi T, et al. (2003) TNF, TNF receptor type 1, and allograft inflammatory factor-1 gene polymorphisms in Japanese patients with type 1 diabetes. Hum Immunol 64: 302-309.
44. Shtauvere-Brameus A, Dabadghao P, Rumba I, Sanjeevi CB (2002) Tumor necrosis factor-alpha allele 2 shows an association with insulin-dependent diabetes mellitus in Latvians. Ann N Y Acad Sci 958: 357-361.

45. Deng GY, Maclaren NK, Huang HS, Zhang LP, She JX (1996) No primary association between the 308 polymorphism in the tumor necrosis factor alpha promoter region and insulin-dependent diabetes mellitus. Hum Immunol 45: 137-142.
46. Cox A, Gonzalez AM, Wilson AG, Wilson RM, Ward JD, et al. (1994) Comparative analysis of the genetic associations of HLA-DR3 and tumour necrosis factor alpha with human IDDM. Diabetologia 37: 500-503.
47. Pociot F, Briant L, Jongeneel CV, Molvig J, Worsaae H, et al. (1993) Association of tumor necrosis factor (TNF) and class II major histocompatibility complex alleles with the secretion of TNF-alpha and TNF-beta by human mononuclear cells: a possible link to insulin-dependent diabetes mellitus. Eur J Immunol 23: 224-231.
48. Badenhoop K, Schwarz G, Bingley P, Trowsdale J, Usadel KH, et al. (1989) TNF-alpha gene polymorphisms: association with type I (insulin-dependent) diabetes mellitus. J Immunogenet 16: 455-460.
49. Noble JA, Valdes AM, Cook M, Klitz W, Thomson G, et al. (1996) The role of HLA class II genes in insulin-dependent diabetes mellitus: molecular analysis of 180 Caucasian, multiplex families. Am J Hum Genet 59: 1134-1148.
50. Valdes AM, Noble JA, Genin E, Clerget-Darpoux F, Erlich HA, et al. (2001) Modeling of HLA class II susceptibility to Type I diabetes reveals an effect associated with DPB1. Genet Epidemiol 21: 212-223.
51. Noble JA, Valdes AM, Bugawan TL, Apple RJ, Thomson G, et al. (2002) The HLA class I A locus affects susceptibility to type 1 diabetes. Hum Immunol 63: 657-664.
52. Valdes AM, Erlich HA, Noble JA (2005) Human leukocyte antigen class I B and C loci contribute to Type 1 Diabetes (T1D) susceptibility and age at T1D onset. Hum Immunol 66: 301-313.
53. Steenkiste A, Valdes AM, Feolo M, Hoffman D, Concannon P, et al. (2007) 14th International HLA and Immunogenetics Workshop: report on the HLA component of type 1 diabetes. Tissue Antigens 69 Suppl 1: 214-225.
54. Cucca F, Lampis R, Congia M, Angius E, Nutland S, et al. (2001) A correlation between the relative predisposition of MHC class II alleles to type 1 diabetes and the structure of their proteins. Hum Mol Genet 10: 2025-2037.
55. Baschal EE, Aly TA, Babu SR, Fernando MS, Yu L, et al. (2007) HLA-DPB1*0402 protects against type 1A diabetes autoimmunity in the highest risk DR3-DQB1*0201/DR4-DQB1*0302 DAISY population. Diabetes 56: 2405-2409.
