# Supplementary material for: Defining the Role of the MHC in Autoimmunity: A Review and Pooled Analysis
Source: PLoS Genet. 2008 Apr 25;4(4):e1000024. doi: 10.1371/journal.pgen.1000024 (PMC2291482; doi:10.1371/journal.pgen.1000024)
Supplement: Dataset S3 — Review and pooled analysis of MHC association with systemic lupus erythematosus. (0.03 MB PDF) [file pgen.1000024.s006.pdf]

Data S3

Systemic lupus erythematosus

Systemic lupus erythematosus (SLE, MIM 152700) is the prototypic, multi-system autoimmune disease primarily affecting women of child bearing age. The aetiology of the disease is not yet understood but genetic, hormonal and environmental influences contribute to disease susceptibility. The worldwide prevalence of SLE is estimated at between 12 and 64 cases per 100,000 individuals, with a 10:1 female gender bias [1]. There is a 4-5 fold higher prevalence in non-European (Afro-Caribbean, Indo-Asian) compared with European populations [1-5]). Lupus is characterized by the production of pathogenic IgG autoantibodies to a wide spectrum of nuclear (dsDNA, histones) and cell surface antigens (antiphospholipid antibodies). The clinical manifestations of the disease are diverse and result from inflammation and damage in a variety of organs. Lupus commonly presents with arthralgia, fatigue and rash but individuals may manifest serious and life-threatening complications due to renal, cerebral and cardiopulmonary involvement.

The human MHC was first shown to be associated with SLE in 1971 when lupus probands were found to be enriched for the class I alleles, *HL-A8* (now known as *HLA-B8*) and *HLA-W15* (now known as *HLA-B15*) when compared with healthy controls [6,7]. Subsequent case-control studies focused on the classical class I and class II genes as well as the complement *C4* locus within the class III region given the association of lupus with C4 deficiency. Interestingly, the MHC has only been significantly linked to SLE in one of the twelve genome-wide linkage scans [8,9] with supporting evidence from three further studies [10-12]. However, a recent meta-analysis of linkage studies in

lupus has demonstrated evidence of significant linkage at *6p21* [13]. There is also clear evidence from approximately 100 case-control association studies that the MHC plays an important role in SLE. However, the majority of these studies have failed to encompass the entire locus and have been undertaken in small ethnically diverse cohorts utilizing a limited number of genetic markers.

The most consistent HLA associations with SLE reside with the class II genes, *HLA-DR3* (*DRB1*0301*) and *-DR2* (*DRB1*1501*) and their respective haplotypes in predominantly white populations [14]. Studies in non-white populations are inconsistent. For instance, investigation of the *HLA-DR* locus in the LUMINA study [15] revealed increased frequency of *DRB1*0301* in Caucasians and Hispanics from Texas. *DRB1*0801* was increased in Hispanics from Texas but not Puerto Rico. African Americans in the same study were found to have higher frequencies of *DRB1*1503* (*DR2*) compared to matched controls. These populations demonstrated no association with *DRB1*1501*. Other studies in African Americans have revealed no association of *DRB1* alleles with SLE [16,17]. An association with *HLA-DR4* was shown in the only study in North Indians [18]. A number of other studies in lupus have demonstrated associations among Mexican [19], Tunisian [20], Korean [21] and Thai [22] populations with *DRB1*15*; Mexican and Tunisian patients with *DRB1*0301* and *DRB3*01/03* in Jamaicans [22].

One might expect a close association between class II alleles and autoantibody subsets in lupus if these are indeed the causal variants. A variety of *HLA-DR* and *-DQ* alleles, have been associated with autoantibody subsets in ethnically diverse lupus populations. The strongest associations

have been demonstrated between anti-Ro/La antibodies and *DR3* and *DQ2* (*DQB1*0201*) which are in strong linkage disequilibrium [23-27]. Studies of individuals with the antiphospholipid syndrome and antiphospholipid antibodies in lupus show predominant association with the *DR4/DQ8* (*DQB1*0302*) haplotype as well as other class II alleles [28-30].

The MHC class I specificities, *A1* and *B8*, have been linked with SLE. However these alleles reside on the disease-associated *DR3* haplotype, AH8.1, and this effect most likely results from linkage disequilibrium.

Despite the fact that the class III region is the most gene-dense in the genome, only complement *C4* and *TNF* polymorphisms have been studied in any detail in lupus. Inherited (and acquired) deficiencies of the early classical complement components, C2, C4A, C4B, encoded within this region are associated with the development of lupus although penetrance is not complete [31]. The presence of *C4A* and *C4B* null alleles (*C4A*Q0* and *C4B*Q0*) which result in partial C4 deficiency have been associated with lupus [32,33]. However, these alleles are in strong linkage disequilibrium with specific ancestral haplotypes: *A1-B8-TNF-308A-C4A*Q0-DRB1*0301* (AH 8.1) and *A30-B18-C4B*Q0-DRB1*0301* (the “Basque” haplotype), so to date it has not been possible to establish the identity of the causal allele(s) located within these extended haplotypes in lupus. The *C4* null allele associations seem to reflect the prevalent *DRB1*0301* haplotype in the population under study. In southern European populations the *C4B* null allele in LD with the *A30-B18-DRB1*0301* haplotype shows association with SLE [33-35] while the *C4A* null allele in LD with the *A1-B8-DRB1*0301* haplotype is associated with lupus in northern European and north American populations. Two methods

have been used to determine whether the *C4* null allele association in lupus is causal or secondary to LD. One strategy involves the study of patients with lupus who do not bear *DR3* haplotypes for association with null alleles, while the second examines ethnically diverse populations in whom distinct extended haplotypes are observed. At present these data are inconsistent and require clarification by examining large lupus cohorts of defined ethnicity whilst accounting for patterns of LD. [20,31,36-38].

A role for another class III gene, tumour necrosis factor alpha (*TNF*), in SLE was suggested by McDevitt in 1988 following the observation that the lupus-prone New Zealand F1 mouse hybrid exhibits constitutively low *TNF* expression [39]. Recently the development of autoimmunity in patients treated with TNF-alpha antagonists has also stimulated interest in the possible role of TNF in SLE [40-42]. Case control studies have examined promoter single nucleotide polymorphisms (SNPs) and microsatellite polymorphisms at the *TNF* locus. Some studies have shown associations with certain *TNF* alleles [43-45]. However, the limited number of polymorphisms genotyped and the strong linkage disequilibrium between certain *TNF* alleles and the *B8-DR3* haplotype again restricts interpretation of these data.

In 2002, Graham et al [46] used a different strategy, that of family-based association to screen the human MHC region in lupus, albeit at low density. This analysis identified three microsatellite-inferred risk haplotypes in Caucasian lupus families: *DRB1*1501/DQB1*0602*, *DRB1*0301/DQB1*0201*, and *DRB1*0801/DQB1*0402*. Further analysis of ancestral recombinants could only delimit the disease associated region to 1 Mb of the MHC encompassing class II and class III.

Taking the above into account it is not surprising that our pooled analysis demonstrates predominant association with variants linked to *DR3* and *DR2*-bearing ancestral haplotypes in SLE (Figure 1). The strongest associations reside within the *DR3* haplotypes, *B8-DRB1*0301* and *B18-DRB1*0301*; specifically the majority of alleles map to the *B8-DRB1*0301* haplotype (*A1, B8, TNFb3, TNFa2, TNF-308A, TNFd1, C4A*Q0, DRB1*0301, DQA1*0501, DQB1*0201*). Half of the 26 significant positive associations lie on these two *DR3* haplotypes and display OR ranging from 1.5 to 2.5. Four of the remaining associations, *DR2*, *DR15, DRB1*1501*, and *DQB1*0602*, map to the *DR2/1501* haplotype, each with odds ratios of approximately 1.7. The association with another *DR2* subtype, *DRB1*1503* is seen in African Americans only, while the *DRB1*1602* allele is observed Mexican Mestizo, Thai, and Bulgarian populations. The observed *DRB1*0401* signal principally arises from Mexican Mestizo and Hispanic cohorts in whom this allele is uncommon (frequency ~1%). This association has not been well described and warrants further investigation. Two further class II alleles, *HLA-DQA1*0401* and *HLA-DQB1*0402*, reside on a *DR8* haplotype which is infrequent in Caucasian populations (frequency ~2%). The significance of the remaining associations with *HLA-B27* (in LD with *DRB1*0103, 0401, 0701* [47]) and the *TNF* microsatellites, *TNFb6, TNFa3, TNFa12* is unclear given the relatively small study numbers and wide confidence intervals.

This pooled analysis highlights the importance of polymorphism within *HLA-DR3*-containing haplotypes in lupus susceptibility. The remaining association

signals largely arise from the classical class II alleles *HLA-DRB1*, *HLA-DQA1* and *HLA-DQB1* and interestingly show evidence of population specificity.

REFERENCES

1. Hochberg MC (1997) The epidemiology of systemic lupus erythematosus. In: Wallace DJ, Hahn BH, editors. Dubois' Lupus Erythematosus. Baltimore: Williams and Wilkins.
2. Samanta A, Roy S, Feehally J, Symmons DP (1992) The prevalence of diagnosed systemic lupus erythematosus in whites and Indian Asian immigrants in Leicester city, UK. Br J Rheumatol 31: 679-682.
3. Johnson AE, Gordon C, Palmer RG, Bacon PA (1995) The prevalence and incidence of systemic lupus erythematosus in Birmingham, England. Relationship to ethnicity and country of birth. Arthritis Rheum 38: 551-558.
4. Molokhia M, Hoggart C, Patrick AL, Shriver M, Parra E, et al. (2003) Relation of risk of systemic lupus erythematosus to west African admixture in a Caribbean population. Hum Genet 112: 310-318.
5. Molokhia M, McKeigue PM, Cuadrado M, Hughes G (2001) Systemic lupus erythematosus in migrants from west Africa compared with Afro-Caribbean people in the UK. Lancet 357: 1414-1415.
6. Grumet FC, Coukell A, Bodmer JG, Bodmer WF, McDevitt HO (1971) Histocompatibility (HL-A) antigens associated with systemic lupus erythematosus. A possible genetic predisposition to disease. N Engl J Med 285: 193-196.
7. Waters H, Konrad P, Walford RL (1971) The distribution of HL-A histocompatibility factors and genes in patients with systemic lupus erythematosus. Tissue Antigens 1: 68-73.
8. Gaffney PM, Kearns GM, Shark KB, Ortmann WA, Selby SA, et al. (1998) A genome-wide search for susceptibility genes in human systemic lupus erythematosus sib-pair families. Proc Natl Acad Sci U S A 95: 14875-14879.
9. Gaffney PM, Ortmann WA, Selby SA, Shark KB, Ockenden TC, et al. (2000) Genome screening in human systemic lupus erythematosus: results from a second Minnesota cohort and combined analyses of 187 sib-pair families. Am J Hum Genet 66: 547-556.
10. Shai R, Quismorio FP, Jr., Li L, Kwon OJ, Morrison J, et al. (1999) Genome-wide screen for systemic lupus erythematosus susceptibility genes in multiplex families. Hum Mol Genet 8: 639-644.
11. Lindqvist AK, Steinsson K, Johanneson B, Kristjansdottir H, Arnasson A, et al. (2000) A susceptibility locus for human systemic lupus erythematosus (hSLE1) on chromosome 2q. J Autoimmun 14: 169-178.
12. Gray-McGuire C, Moser KL, Gaffney PM, Kelly J, Yu H, et al. (2000) Genome scan of human systemic lupus erythematosus by regression modeling: evidence of linkage and epistasis at 4p16-15.2. Am J Hum Genet 67: 1460-1469.

13. Forabosco P, Gorman JD, Cleveland C, Kelly JA, Fisher SA, et al. (2006) Meta-analysis of genome-wide linkage studies of systemic lupus erythematosus. Genes Immun 7: 609-614.
14. Tsao BP (2004) Update on human systemic lupus erythematosus genetics. Curr Opin Rheumatol 16: 513-521.
15. Uribe AG, McGwin G, Jr., Reveille JD, Alarcon GS (2004) What have we learned from a 10-year experience with the LUMINA (Lupus in Minorities; Nature vs. nurture) cohort? Where are we heading? Autoimmun Rev 3: 321-329.
16. Reveille JD, Schrohenloher RE, Acton RT, Barger BO (1989) DNA analysis of HLA-DR and DQ genes in American blacks with systemic lupus erythematosus. Arthritis Rheum 32: 1243-1251.
17. Howard PF, Hochberg MC, Bias WB, Arnett FC, Jr., McLean RH (1986) Relationship between C4 null genes, HLA-D region antigens, and genetic susceptibility to systemic lupus erythematosus in Caucasian and black Americans. Am J Med 81: 187-193.
18. Mehra NK, Pande I, Taneja V, Uppal SS, Saxena SP, et al. (1993) Major histocompatibility complex genes and susceptibility to systemic lupus erythematosus in northern India. Lupus 2: 313-314.
19. Cortes LM, Baltazar LM, Lopez-Cardona MG, Olivares N, Ramos C, et al. (2004) HLA class II haplotypes in Mexican systemic lupus erythematosus patients. Hum Immunol 65: 1469-1476.
20. Ayed K, Gorgi Y, Ayed-Jendoubi S, Bardi R (2004) The involvement of HLA -DRB1*, DQA1*, DQB1* and complement C4A loci in diagnosing systemic lupus erythematosus among Tunisians. Ann Saudi Med 24: 31-35.
21. Lee HS, Chung YH, Kim TG, Kim TH, Jun JB, et al. (2003) Independent association of HLA-DR and FCgamma receptor polymorphisms in Korean patients with systemic lupus erythematosus. Rheumatology (Oxford) 42: 1501-1507.
22. Smikle M, Christian N, DeCeulaer K, Barton E, Roye-Green K, et al. (2002) HLA-DRB alleles and systemic lupus erythematosus in Jamaicans. South Med J 95: 717-719.
23. Schur PH (1995) Genetics of systemic lupus erythematosus. Lupus 4: 425-437.
24. Logar D, Vidan-Jeras B, Dolzan V, Bozic B, Kveder T (2002) The contribution of HLA-DQB1 coding and QBP promoter alleles to anti-Ro alone autoantibody response in systemic lupus erythematosus. Rheumatology (Oxford) 41: 305-311.
25. Azizah MR, Ainoi SS, Kuak SH, Kong NC, Normaznah Y, et al. (2001) The association of the HLA class II antigens with clinical and autoantibody expression in Malaysian Chinese patients with systemic lupus erythematosus. Asian Pac J Allergy Immunol 19: 93-100.
26. Miyagawa S, Shinohara K, Nakajima M, Kidoguchi K, Fujita T, et al. (1998) Polymorphisms of HLA class II genes and autoimmune responses to Ro/SS-A-La/SS-B among Japanese subjects. Arthritis Rheum 41: 927-934.
27. Galeazzi M, Sebastiani GD, Morozzi G, Carcassi C, Ferrara GB, et al. (2002) HLA class II DNA typing in a large series of European patients

with systemic lupus erythematosus: correlations with clinical and autoantibody subsets. Medicine (Baltimore) 81: 169-178.
28. Arnett FC, Olsen ML, Anderson KL, Reveille JD (1991) Molecular analysis of major histocompatibility complex alleles associated with the lupus anticoagulant. J Clin Invest 87: 1490-1495.
29. Arnett FC, Thiagarajan P, Ahn C, Reveille JD (1999) Associations of anti-beta2-glycoprotein I autoantibodies with HLA class II alleles in three ethnic groups. Arthritis Rheum 42: 268-274.
30. Galeazzi M, Sebastiani GD, Tincani A, Piette JC, Allegri F, et al. (2000) HLA class II alleles associations of anticardiolipin and anti-beta2GPI antibodies in a large series of European patients with systemic lupus erythematosus. Lupus 9: 47-55.
31. Pickering MC, Walport MJ (2000) Links between complement abnormalities and systemic lupus erythematosus. Rheumatology (Oxford) 39: 133-141.
32. Pickering MC, Perraudeau M, Walport MJ (2000) HLA and Systemic Vasculitides, Systemic Lupus Erythematosus and Sjogren's Syndrome. In: Lechler R, Warrens A, editors. HLA in Health and Disease. Second ed: Academic Press. pp. 327-364.
33. Naves M, Hajeer AH, Teh LS, Davies EJ, Ordi-Ros J, et al. (1998) Complement C4B null allele status confers risk for systemic lupus erythematosus in a Spanish population. Eur J Immunogenet 25: 317-320.
34. Gomez-Reino JJ, Martinez-Laso J, Vicario JL, Paz-Artal E, Aragon A, et al. (1991) Immunogenetics of systemic lupus erythematosus in Spanish patients: differential HLA markers. Immunobiology 182: 465-471.
35. De Juan D, Martin-Villa JM, Gomez-Reino JJ, Vicario JL, Corell A, et al. (1993) Differential contribution of C4 and HLA-DQ genes to systemic lupus erythematosus susceptibility. Hum Genet 91: 579-584.
36. Batchelor JR, Fielder AH, Walport MJ, David J, Lord DK, et al. (1987) Family study of the major histocompatibility complex in HLA DR3 negative patients with systemic lupus erythematosus. Clin Exp Immunol 70: 364-371.
37. Hartung K, Baur MP, Coldewey R, Fricke M, Kalden JR, et al. (1992) Major histocompatibility complex haplotypes and complement C4 alleles in systemic lupus erythematosus. Results of a multicenter study. J Clin Invest 90: 1346-1351.
38. Yang Y, Chung EK, Wu YL, Savelli SL, Nagaraja HN, et al. (2007) Gene copy-number variation and associated polymorphisms of complement component C4 in human systemic lupus erythematosus (SLE): low copy number is a risk factor for and high copy number is a protective factor against SLE susceptibility in European Americans. Am J Hum Genet 80: 1037-1054.
39. Jacob CO, McDevitt HO (1988) Tumour necrosis factor-alpha in murine autoimmune 'lupus' nephritis. Nature 331: 356-358.
40. Charles PJ, Smeenk RJ, De Jong J, Feldmann M, Maini RN (2000) Assessment of antibodies to double-stranded DNA induced in rheumatoid arthritis patients following treatment with infliximab, a monoclonal antibody to tumor necrosis factor alpha: findings in open-

label and randomized placebo-controlled trials. Arthritis Rheum 43: 2383-2390.
41. Shakoor N, Michalska M, Harris CA, Block JA (2002) Drug-induced systemic lupus erythematosus associated with etanercept therapy. Lancet 359: 579-580.
42. Vermeire S, Noman M, Van Assche G, Baert F, Van Steen K, et al. (2003) Autoimmunity associated with anti-tumor necrosis factor alpha treatment in Crohn's disease: a prospective cohort study. Gastroenterology 125: 32-39.
43. Wilson AG, Duff GW (1996) Genetics of tumour necrosis factor in systemic lupus erythematosus. Lupus 5: 87-88.
44. Hajeer AH, Worthington J, Davies EJ, Hillarby MC, Poulton K, et al. (1997) TNF microsatellite a2, b3 and d2 alleles are associated with systemic lupus erythematosus. Tissue Antigens 49: 222-227.
45. van der Linden MW, van der Slik AR, Zanelli E, Giphart MJ, Pieterman E, et al. (2001) Six microsatellite markers on the short arm of chromosome 6 in relation to HLA-DR3 and TNF-308A in systemic lupus erythematosus. Genes Immun 2: 373-380.
46. Graham RR, Ortmann WA, Langefeld CD, Jawaheer D, Selby SA, et al. (2002) Visualizing human leukocyte antigen class II risk haplotypes in human systemic lupus erythematosus. Am J Hum Genet 71: 543-553.
47. Cattley SK, Williamson JF, Tay GK, Martinez OP, Gaudieri S, et al. (2000) Further characterization of MHC haplotypes demonstrates conservation telomeric of HLA-A: update of the 4AOH and 10IHW cell panels. Eur J Immunogenet 27: 397-426.
