# Supplementary material for: Defining the Role of the MHC in Autoimmunity: A Review and Pooled Analysis
Source: PLoS Genet. 2008 Apr 25;4(4):e1000024. doi: 10.1371/journal.pgen.1000024 (PMC2291482; doi:10.1371/journal.pgen.1000024)
Supplement: Dataset S4 — Review and pooled analysis of MHC association with inflammatory bowel disease. (0.03 MB PDF) [file pgen.1000024.s007.pdf]

Data S4

Inflammatory bowel disease: Ulcerative Colitis and Crohn's disease

Crohn's disease (CD) and ulcerative colitis (UC) are related inflammatory diseases of the gastrointestinal tract commonly known as inflammatory bowel diseases (IBD, MIM 266600).These diseases show a peak incidence in early adulthood and affect approximately 1-2 individuals per thousand. Clinically, CD is characterized by a discontinuous transmural inflammation affecting any portion of the gastrointestinal tract, but most commonly the terminal ileum; while UC is characterized by inflammation of the colonic mucosa, extending to a variable extent, from the rectum to the proximal colon.

Several independent genome-wide scans in both CD and UC have shown evidence of linkage to the MHC (also known as the *IBD3* locus) [1-7]. A recent meta-analysis of genome-wide linkage data has confirmed the importance of this region in IBD [8]. Interestingly, it has been suggested that this region may exert a greater effect in susceptibility to UC rather than CD. Specifically, based on estimates of empirical risk and monozygotic twin concordance rate, the genetic contribution of the *IBD3* region to disease has been estimated as 60-100% for UC and closer to 10% for CD [9].

Over the past 35 years, over 100 association studies have investigated the role of the MHC in determining IBD susceptibility and clinical expression. These early studies focused on the classical HLA genes, particularly the *HLA-DR* alleles. The role of *TNF* promoter polymorphisms in IBD susceptibility has been the focus of recent interest given the successful use of the TNF-alpha antagonist, infliximab, in the treatment of active IBD. Initial studies of the MHC in IBD have shown inconsistent associations and suffered from a lack of

power due to small sample size and investigation of a limited number of loci. To gain further insight into the role of the class I genes *HLA-A* and *HLA-B*, Biemond et al. [10] undertook a meta-analysis of 12 CD and 14 UC association studies published between 1972 and 1982. This study confirmed the presence of predisposing and protective alleles in the class I region, including the risk alleles *HLA-A2* and *HLA-B18* for CD and *HLA-B27, HLA-B35* and *HLA-B5* for UC. Similarly, Stokkers et al [11] undertook a meta-analysis of 19 UC and 17 CD studies published between 1980 and 1998 for the class II genes, *HLA-DR* and *-DQ* in IBD. This study confirmed the presence of several predisposing and protective alleles in the class II region, including the risk alleles *HLA-DR7, HLA-DQ4* and *HLA-DRB3*0301* for CD and *HLA-DR9, HLA-DRB1*1502* and *HLA-DRB1*0103* for UC. Subsequent studies have provided supporting evidence for some, but not all of these observations, as well as further describing the association of specific *HLA-DRB1* alleles with clinical sub-phenotypes.

*HLA-DRB1*1502* and *HLA-DRB1*0103* have shown consistent association with UC [11-18]. *HLA-DR2* and its sub-specificity *HLA-DR15*, both of which encompass *HLA-DRB1*1502*, also predispose to UC [11] most likely as a result of the latter association. *HLA-DRB1*1502* predisposes to disease in different populations including Japanese UC cohorts where the allele is highly prevalent (20-25%) [13,15,17,18], and European populations where it is rare (less than 1%) [19]. The class I allele *HLA-B*52* [14,18], found in LD with *DRB1*1502*, has also shown association with disease. It is presently unclear where the primary association lies given the long range conservation of this haplotype. *HLA-DRB1*0103* represents the most reproducible association

observed to date in UC [11,12,14,20-23]. In recent studies, this allele has been associated with a more aggressive disease phenotype, a shorter mean time to surgery, as well as the presence of extra-intestinal manifestations [14,20,21]. Microsatellite polymorphisms of the class I-related gene, *MICA*, have also been investigated in IBD patients, and non-replicated associations were observed for 3 different alleles *MICA*007*, *MICA5.1* and *MICA6* with UC in Caucasian, Chinese and Japanese populations respectively [18,24,25]. The *MICA*007* allele was in LD with *HLA-B27*, while the *MICA6* association in Japanese UC was found to be secondary to *HLA-B52* [18,24]. Finally, investigation of the class III locus in UC has largely centered on the role of *TNF* promoter polymorphisms (as discussed above). So far the results of these studies have not proven to be conclusive.

The current pooled analysis includes 37 UC and 40 CD studies, of which 9 UC and 11 CD studies are common to the previously published study by Stokkers et al [11].

The main association signals (7 out of 13) shown for UC in our pooled analysis arise from *HLA-DRB1*0103* and *HLA-DRB1*1502* haplotypes and substantiate the current literature base (Figure 2). The greatest OR in the dataset are observed with the individual alleles *HLA-DRB1*0103* (OR 4.6), encompassed within the *HLA-DR1* specificity (OR 1.4), *HLA-DRB1*1502* (OR 3.2) and *HLA-B52* (OR 3.3) which is in LD with *HLA-DRB1*1502*. The broad specificities, *HLA-DR2* and *HLA-DR15* also show association. Our results also demonstrate a weak predisposing effect of the microsatellite allele *MICA5.1* in UC. Other novel associations are demonstrated for *HLA-DR5* (and its sub-

specificities *HLA-DR11* and *-DR12*), *HLA-A19* and *HLA-A24* (in LD with *DRB1*1502* and other alleles including *HLA-DRB1*0405*).

In contrast to UC, four separate *HLA-DRB* alleles show reproducible association with CD: *HLA-DRB1*07, HLA-DRB1*0103, HLA-DRB1*04* and *HLA-DRB3*0301* [11]. The most consistently replicated association between the MHC and CD is that with *HLA-DRB1*07* [11,26-28]; specifically, *HLA-DRB1*0701* is associated with ileal disease [26-28]. *DRB1*0103* is associated with susceptibility to CD as well as UC [11,12,26-29] and shows sub-phenotype specificity to colonic CD [26-28]. However, the low prevalence of *DRB1*0103* (less than 2% in Europeans) restricts its usefulness in predicting clinical outcome. Several studies have reported weak association of *HLA-DRB1*04* [11,13,27,30,31] with CD. In particular, the subtypes *HLA-DRB1*0405* and *HLA-DRB1*0410* and their linked *HLA-DQ* alleles, *DQB1*0401* and *DQB1*0402* respectively, are associated with CD predominantly in patients of Japanese origin. A small number of studies have reported association of *HLA-DRB3*0301* with CD [11,26]. However, the *HLA-DRB3* locus has not been subject to a thorough investigation in CD since it is present in less than 50% of European and white North American populations due to haplotype-specific expression. Disease association is further confounded by the predisposing *HLA-DRB1*1302* and *HLA-Cw*0802* alleles which are in tight LD with *HLA-DRB3*0301* [26]. The majority of studies examining polymorphisms in the *TNF* promoter have yielded contradictory results, with only the *TNF-857* promoter polymorphism showing replicated association with susceptibility to CD [32-36].

The current pooled analysis includes 37 UC and 40 CD studies, of which 9 UC and 11 CD studies are common to the previously published study by Stokkers et al [11].

Fourteen of the 22 significant association signals in CD map to haplotypes previously shown to predispose to disease, specifically *HLA-DRB1*0103, HLA-DR4, HLA-DR7* and *HLA-DRB3*0301* (Figure 2). The strongest association is observed with *HLA-DRB1*0410* (OR 3.9). The *HLA-DR4* specificity encompasses *HLA-DRB1*0410* and *HLA-DRB1*0405*, all of which demonstrate association with susceptibility to CD. Similarly, *HLA-DQB1*0401* and *HLA-DQB1*0402*, subtypes of *HLA-DQB1*04* and found in LD with *HLA-DRB1*0405* and *HLA-DRB1*0410* respectively, also show significant association with CD. As described in previous studies, the association signal at *HLA-DRB1*0410* in our pooled analysis largely derives from Japanese patients: of all CD study cases bearing *HLA-DRB1*0410* in this pooled analysis 60% were of Japanese origin, while CD patients of Japanese origin represent only 18% of the overall CD sample base. The *HLA-DRB3*0301* linked alleles, *HLA-Cw8*, *HLA-DRB1*1302* (together with *HLA-DQB1*0604* found in LD with *1302*), show association in this study, however we find no association specifically with *HLA-DRB3*0301* as the relevant studies did not fulfill our inclusion criteria. We demonstrate a robust signal with *HLA-DRB1*0103* allele, previously associated with colonic CD. *HLA-DRB1*07* reported to be weakly associated with CD, also shows a weak signal in our study with narrow CI. We also confirm previously reported association signals with *HLA-B18* and *HLA-B21* class I alleles, and identify novel associations to

HLA-DR6 (encompassing *HLA-DRB1*1401*), *HLA-DR8* (including *HLA-DRB1*0802* and *0803*), and *HLA-DR10* class II subtypes in CD (OR~1.5).

We confirm previous reports of association of the *TNF* promoter polymorphism, *TNF-857T* with CD in Japanese populations and further show association with *TNF-1031C* and *TNF-863A* again in Japanese cohorts; all three SNPs show modest association with OR from 1.3 to 1.5. In contrast, case-control and family-based studies in Caucasian CD show association with the common *TNF-857C* allele suggesting that the polymorphism itself is not causal but shows association secondary to LD [32,34,35].

In summary, the current pooled analysis confirms the previously reported MHC associations in IBD, but also identifies several novel potentially predisposing alleles. The shared association of *DRB1*0103* with UC and colonic CD supports the notion of a common molecular basis for the colonic IBD phenotype. It has been suggested that the MHC plays a stronger role in susceptibility to UC rather than CD, however, given the large number of relatively low risk variants in CD, it is possible that the overall effect of MHC risk in CD is quite sizeable, albeit genetically heterogeneous.

REFERENCES

1. Hampe J, Schreiber S, Shaw SH, Lau KF, Bridger S, et al. (1999) A genomewide analysis provides evidence for novel linkages in inflammatory bowel disease in a large European cohort. Am J Hum Genet 64: 808-816.
2. Ma Y, Ohmen JD, Li Z, Bentley LG, McElree C, et al. (1999) A genome-wide search identifies potential new susceptibility loci for Crohn's disease. Inflamm Bowel Dis 5: 271-278.
3. Satsangi J, Parkes M, Louis E, Hashimoto L, Kato N, et al. (1996) Two stage genome-wide search in inflammatory bowel disease provides evidence for susceptibility loci on chromosomes 3, 7 and 12. Nat Genet 14: 199-202.

4. Cho JH, Nicolae DL, Gold LH, Fields CT, LaBuda MC, et al. (1998) Identification of novel susceptibility loci for inflammatory bowel disease on chromosomes 1p, 3q, and 4q: evidence for epistasis between 1p and IBD1. Proc Natl Acad Sci U S A 95: 7502-7507.
5. Rioux JD, Silverberg MS, Daly MJ, Steinhart AH, McLeod RS, et al. (2000) Genomewide search in Canadian families with inflammatory bowel disease reveals two novel susceptibility loci. Am J Hum Genet 66: 1863-1870.
6. Duerr RH, Barmada MM, Zhang L, Pfutzer R, Weeks DE (2000) High-density genome scan in Crohn disease shows confirmed linkage to chromosome 14q11-12. Am J Hum Genet 66: 1857-1862.
7. Hugot JP, Laurent PP, Gower RC, Olson JM, Lee JC, et al. (1996) Mapping of a susceptibility locus for Crohn's disease on chromosome 16. Nature 379: 821-823.
8. van Heel DA, Fisher SA, Kirby A, Daly MJ, Rioux JD, et al. (2004) Inflammatory bowel disease susceptibility loci defined by genome scan meta-analysis of 1952 affected relative pairs. Hum Mol Genet 13: 763-770.
9. Satsangi J, Welsh KI, Bunce M, Julier C, Farrant JM, et al. (1996) Contribution of genes of the major histocompatibility complex to susceptibility and disease phenotype in inflammatory bowel disease. Lancet 347: 1212-1217.
10. Biemond I, Burnham WR, D'Amaro J, Langman MJ (1986) HLA-A and -B antigens in inflammatory bowel disease. Gut 27: 934-941.
11. Stokkers PC, Reitsma PH, Tytgat GN, van Deventer SJ (1999) HLA-DR and -DQ phenotypes in inflammatory bowel disease: a meta-analysis. Gut 45: 395-401.
12. Trachtenberg EA, Yang H, Hayes E, Vinson M, Lin C, et al. (2000) HLA class II haplotype associations with inflammatory bowel disease in Jewish (Ashkenazi) and non-Jewish caucasian populations. Hum Immunol 61: 326-333.
13. Yoshitake S, Kimura A, Okada M, Yao T, Sasazuki T (1999) HLA class II alleles in Japanese patients with inflammatory bowel disease. Tissue Antigens 53: 350-358.
14. Ahmad T, Armuzzi A, Neville M, Bunce M, Ling KL, et al. (2003) The contribution of human leucocyte antigen complex genes to disease phenotype in ulcerative colitis. Tissue Antigens 62: 527-535.
15. Futami S, Aoyama N, Honsako Y, Tamura T, Morimoto S, et al. (1995) HLA-DRB1*1502 allele, subtype of DR15, is associated with susceptibility to ulcerative colitis and its progression. Dig Dis Sci 40: 814-818.
16. Myung SJ, Yang SK, Jung HY, Chang HS, Park B, et al. (2002) HLA-DRB1*1502 confers susceptibility to ulcerative colitis, but is negatively associated with its intractability: a Korean study. Int J Colorectal Dis 17: 233-237.
17. Masuda H, Nakamura Y, Tanaka T, Hayakawa S (1994) Distinct relationship between HLA-DR genes and intractability of ulcerative colitis. Am J Gastroenterol 89: 1957-1962.
18. Seki SS, Sugimura K, Ota M, Matsuzawa J, Katsuyama Y, et al. (2001) Stratification analysis of MICA triplet repeat polymorphisms and HLA

antigens associated with ulcerative colitis in Japanese. Tissue Antigens 58: 71-76.
19. Ahmad T, Marshall SE, Jewell D (2006) Genetics of inflammatory bowel disease: the role of the HLA complex. World J Gastroenterol 12: 3628-3635.
20. Yamamoto-Furusho JK, Uscanga LF, Vargas-Alarcon G, Ruiz-Morales JA, Higuera L, et al. (2003) Clinical and genetic heterogeneity in Mexican patients with ulcerative colitis. Hum Immunol 64: 119-123.
21. Bouma G, Crusius JB, Garcia-Gonzalez MA, Meijer BU, Hellemans HP, et al. (1999) Genetic markers in clinically well defined patients with ulcerative colitis (UC). Clin Exp Immunol 115: 294-300.
22. Roussomoustakaki M, Satsangi J, Welsh K, Louis E, Fanning G, et al. (1997) Genetic markers may predict disease behavior in patients with ulcerative colitis. Gastroenterology 112: 1845-1853.
23. Fernandez-Arquero M, Arroyo R, Rubio A, Martin C, Vigil P, et al. (1999) Primary association of a TNF gene polymorphism with susceptibility to multiple sclerosis. Neurology 53: 1361-1363.
24. Orchard TR, Dhar A, Simmons JD, Vaughan R, Welsh KI, et al. (2001) MHC class I chain-like gene A (MICA) and its associations with inflammatory bowel disease and peripheral arthropathy. Clin Exp Immunol 126: 437-440.
25. Ding Y, Xia B, Lu M, Zhang Y, Li J, et al. (2005) MHC class I chain-related gene A-A5.1 allele is associated with ulcerative colitis in Chinese population. Clin Exp Immunol 142: 193-198.
26. Ahmad T, Armuzzi A, Bunce M, Mulcahy-Hawes K, Marshall SE, et al. (2002) The molecular classification of the clinical manifestations of Crohn's disease. Gastroenterology 122: 854-866.
27. Newman B, Silverberg MS, Gu X, Zhang Q, Lazaro A, et al. (2004) CARD15 and HLA DRB1 alleles influence susceptibility and disease localization in Crohn's disease. Am J Gastroenterol 99: 306-315.
28. Fernandez L, Mendoza JL, Martinez A, Urcelay E, Fernandez-Arquero M, et al. (2004) IBD1 and IBD3 determine location of Crohn's disease in the Spanish population. Inflamm Bowel Dis 10: 715-722.
29. Silverberg MS, Mirea L, Bull SB, Murphy JE, Steinhart AH, et al. (2003) A population- and family-based study of Canadian families reveals association of HLA DRB1*0103 with colonic involvement in inflammatory bowel disease. Inflamm Bowel Dis 9: 1-9.
30. Matake H, Okabe N, Naito S, Yao T (1992) An HLA study on 149 Japanese patients with Crohn's disease. Gastroenterol Jpn 27: 496-501.
31. Nakajima A, Matsuhashi N, Kodama T, Yazaki Y, Takazoe M, et al. (1995) HLA-linked susceptibility and resistance genes in Crohn's disease. Gastroenterology 109: 1462-1467.
32. Tremelling M, Waller S, Bredin F, Greenfield S, Parkes M (2006) Genetic variants in TNF-alpha but not DLG5 are associated with inflammatory bowel disease in a large United Kingdom cohort. Inflamm Bowel Dis 12: 178-184.
33. Negoro K, Kinouchi Y, Hiwatashi N, Takahashi S, Takagi S, et al. (1999) Crohn's disease is associated with novel polymorphisms in the 5'-

flanking region of the tumor necrosis factor gene. Gastroenterology 117: 1062-1068.
34. van Heel DA, Udalova IA, De Silva AP, McGovern DP, Kinouchi Y, et al. (2002) Inflammatory bowel disease is associated with a TNF polymorphism that affects an interaction between the OCT1 and NF(-kappa)B transcription factors. Hum Mol Genet 11: 1281-1289.
35. O'Callaghan NJ, Adams KE, van Heel DA, Cavanaugh JA (2003) Association of TNF-alpha-857C with inflammatory bowel disease in the Australian population. Scand J Gastroenterol 38: 533-534.
36. Fowler EV, Eri R, Hume G, Johnstone S, Pandeya N, et al. (2005) TNFalpha and IL10 SNPs act together to predict disease behaviour in Crohn's disease. J Med Genet 42: 523-528.
