# Supplementary material for: Defining the Role of the MHC in Autoimmunity: A Review and Pooled Analysis
Source: PLoS Genet. 2008 Apr 25;4(4):e1000024. doi: 10.1371/journal.pgen.1000024 (PMC2291482; doi:10.1371/journal.pgen.1000024)
Supplement: Dataset S5 — Review and pooled analysis of MHC association with rheumatoid arthritis. (0.07 MB PDF) [file pgen.1000024.s008.pdf]

Data S5

Rheumatoid arthritis

Rheumatoid arthritis (RA, MIM 180300) is a systemic, chronic inflammatory disorder whose root cause is unclear. The clinical hallmark of RA is an inflammatory arthritis with a predilection for specific diarthrodial (freely movable) joints. The clinical course of RA is extremely variable: some patients suffer mild, self-limiting arthritis while others develop progressive multi-system inflammation with profound morbidity and mortality. Auto-antibodies (Rheumatoid factor (RF) and anti-cyclic citrullinated peptide (CCP)) have proven very useful in predicting which patients will develop severe disease.

RA is the most common form of inflammatory arthritis, with an estimated worldwide prevalence of up to ~1% in the adult population. Females are at greater risk than males for developing the disease, with a female:male ratio of ~2.5:1. While the disease can occur at any age, the peak age on onset is in the 40's, with an increasing incidence with age. To date, only two genes have been convincingly demonstrated to influence risk of RA (*HLA-DRB1* and the tyrosine phosphatase, *PTPN22*).

It has been estimated that the MHC region of the human genome accounts for approximately one-third of the overall genetic component of RA risk [1,2]. Genome-wide linkage scans using both microsatellite [3-7] and single nucleotide polymorphism (SNP) markers [8] have consistently identified this region as important in RA pathogenesis. These genome-wide scans have demonstrated that the MHC confers the largest genetic contribution in RA, and the relative contribution of HLA genes (λ_{HLA}) was found to be ~1.75 [3,5].

Much, but probably not all of the risk attributable to the MHC region is associated with alleles within the *HLA-DRB1* gene. An association between RA and the class II HLA proteins was first noted in the 1970's, when the mixed lymphocyte culture (MLC) type *Dw4* (related to the serological subtype *DR4*) was observed to be more common among patients with RA compared to controls [9,10]. Subsequently, investigation of the molecular diversity of class II proteins (subunits of *HLA-DR, -DQ* and *-DP*) localized the serological *Dw4* subtype to the *HLA-DRB1* gene [11,12]. When the susceptible *DR* subtypes were considered as a group, Gregersen et al noted a shared amino acid sequence at positions 70-74 of the HLA-DRB1 protein [13]. These alleles are now known collectively as "shared epitope" alleles due to the related sequence composition in the third hypervariable region: the susceptibility alleles result in missense amino acid changes, where the shared susceptibility amino acid motif is ^{70}Q/R-K/R-R-A-A^{74}.

Since the initial observation, a large number of population studies have confirmed the association between RA and allelic variants at *HLA-DRB1* [14]. At the level of DNA, the most common (>5% population frequency) *HLA-DRB1* shared epitope susceptibility alleles include **0101, *0401*, and **0404* in individuals of European ancestry, and **0405* and **0901* in individuals of Asian ancestry; less common shared epitope alleles include **0102, *0104, *0408, *0413, *0416, *1001*, and **1402*. Of note, the **0901* allele observed among Asian populations does not strictly conform to the SE amino acid sequence motif (^{70}R-R-R-A-**E**^{74}), and the classic SE alleles may not contribute to risk in African-American and Hispanic-American RA populations [15,16].

While *HLA-DRB1* susceptibility alleles are often considered as a group, the strength of the genetic association to RA susceptibility differs across the *DRB1* alleles. There are at least two classes of *HLA-DRB1* risk alleles (high and moderate). In general, the *DRB1*0401* allele exhibits a high level of risk, with a relative risk (RR) of approximately 3. The *DRB1*0101, *0404, *1001*, and **0901* alleles exhibit a more moderate relative risk in the range of 1.5. These relative risk estimates are for all cases of RA. However, it is becoming increasingly clear that *HLA-DRB1* shared epitope alleles only influence the development of seropositive RA, and more specifically anti-CCP+ RA [17,18]. Collectively, the shared epitope alleles have an odds ratio of over 5 if CCP+ RA patients are compared to matched healthy controls. Because these alleles are quite common in the general population (collectively, allele frequency ~40% in individuals of European ancestry), the attributable risk for SE alleles is quite high.

Several investigators have proposed a refined classification of shared epitope alleles, as this hypothesis alone cannot explain all of the genetic risk attributable to the *HLA-DRB1* locus [19-22]. No consensus has emerged, however. Some of these studies suggest that a protective allele may be in linkage disequilibrium with the *HLA-DRB1* alleles. It has been hypothesized that the presence of an asparagine amino acid at position 70 of the HLA-DRB1 protein (D70) may be associated with protection from the development of RA (once the effect of the shared epitope alleles has been taking into consideration) [23,24].

Several studies suggest that additional genes within the MHC likely contribute to disease susceptibility, once the effect of *HLA-DRB1* has been taken into

consideration [25-29]. For example, an extended haplotype that includes *HLA-DRB1*03* alleles may be associated with RA [25]. The associated haplotype spans ~500kb, and contains MHC class III genes, including the *TNF* locus implicated in other studies [28,30,31]. One study suggests that this association is restricted to CCP-negative patients [18].

Genetic variation at the *HLA-DRB1* gene is clearly associated with rheumatoid arthritis (Figure 2). Our results suggest the existence of at least two classes of risk alleles (high and moderate). The **0401* and **0405* alleles have odds ratios in the range of 3.5, with lower-limit confidence intervals >3.0. The *HLA-B54* allele is in LD with *DR4* alleles (*0405* in particular), which likely explains the association. The next class of *HLA-DRB1* alleles have OR in the 1.5-2.0 range. These alleles include: **0101, *0404, *0901*, and **1001*; additional *DR4* alleles are also included in this group (*Dw14_0404-0408* alleles).
The **0401* allele is the only *HLA-DRB1* allele with amino acid sequence ^{70}Q-K-R-A-A^{74} in the third hypervariable region; the **0401* and **0405* alleles differ at amino acid 71 (where **0405* has an R). The **0405* allele, however, has the identical sequence as the **0404* allele. Thus, if the OR we observe for **0404* and **0405* are indicative of the true OR for these alleles, then a simple molecular model that includes variation at these critical amino acids is insufficient to explain the risk conferred by these alleles. In addition, our results do not support the classification schemes proposed by Gao and Michou [19,22], which differentiate between risk conferred by the **0401* and **0405* alleles.

Our results also support the hypothesis that genetic variation located a significant genomic distance away from the *HLA-DRB1* gene – and thus possibly not in LD with any of the *HLA-DRB1* risk alleles – also appears associated with RA risk. For example, alleles near the *TNF* locus appear associated with RA (e.g., *TNFa13*, OR 1.5).

REFERENCES

1. Deighton CM, Walker DJ, Griffiths ID, Roberts DF (1989) The contribution of HLA to rheumatoid arthritis. Clin Genet 36: 178-182.
2. Rigby AS, Silman AJ, Voelm L, Gregory JC, Ollier WE, et al. (1991) Investigating the HLA component in rheumatoid arthritis: an additive (dominant) mode of inheritance is rejected, a recessive mode is preferred. Genet Epidemiol 8: 153-175.
3. Jawaheer D, Seldin MF, Amos CI, Chen WV, Shigeta R, et al. (2001) A genomewide screen in multiplex rheumatoid arthritis families suggests genetic overlap with other autoimmune diseases. Am J Hum Genet 68: 927-936.
4. Jawaheer D, Seldin MF, Amos CI, Chen WV, Shigeta R, et al. (2003) Screening the genome for rheumatoid arthritis susceptibility genes: a replication study and combined analysis of 512 multicase families. Arthritis Rheum 48: 906-916.
5. Cornelis F, Faure S, Martinez M, Prud'homme JF, Fritz P, et al. (1998) New susceptibility locus for rheumatoid arthritis suggested by a genome-wide linkage study. Proc Natl Acad Sci U S A 95: 10746-10750.
6. MacKay K, Eyre S, Myerscough A, Milicic A, Barton A, et al. (2002) Whole-genome linkage analysis of rheumatoid arthritis susceptibility loci in 252 affected sibling pairs in the United Kingdom. Arthritis Rheum 46: 632-639.
7. Shiozawa S, Hayashi S, Tsukamoto Y, Goko H, Kawasaki H, et al. (1998) Identification of the gene loci that predispose to rheumatoid arthritis. Int Immunol 10: 1891-1895.
8. Amos CI, Chen WV, Lee A, Li W, Kern M, et al. (2006) High-density SNP analysis of 642 Caucasian families with rheumatoid arthritis identifies two new linkage regions on 11p12 and 2q33. Genes Immun.
9. Stastny P (1978) Association of the B-cell alloantigen DRw4 with rheumatoid arthritis. N Engl J Med 298: 869-871.
10. Stastny P, Fink CW (1977) HLA-Dw4 in adult and juvenile rheumatoid arthritis. Transplant Proc 9: 1863-1866.
11. Gregersen PK, Moriuchi T, Karr RW, Obata F, Moriuchi J, et al. (1986) Polymorphism of HLA-DR beta chains in DR4, -7, and -9 haplotypes: implications for the mechanisms of allelic variation. Proc Natl Acad Sci U S A 83: 9149-9153.

12. Gregersen PK, Shen M, Song QL, Merryman P, Degar S, et al. (1986) Molecular diversity of HLA-DR4 haplotypes. Proc Natl Acad Sci U S A 83: 2642-2646.
13. Gregersen PK, Silver J, Winchester RJ (1987) The shared epitope hypothesis. An approach to understanding the molecular genetics of susceptibility to rheumatoid arthritis. Arthritis Rheum 30: 1205-1213.
14. Ollier W, Thomson W (1992) Population genetics of rheumatoid arthritis. Rheum Dis Clin North Am 18: 741-759.
15. McDaniel DO, Alarcon GS, Pratt PW, Reveille JD (1995) Most African-American patients with rheumatoid arthritis do not have the rheumatoid antigenic determinant (epitope). Ann Intern Med 123: 181-187.
16. Teller K, Budhai L, Zhang M, Haramati N, Keiser HD, et al. (1996) HLA-DRB1 and DQB typing of Hispanic American patients with rheumatoid arthritis: the "shared epitope" hypothesis may not apply. J Rheumatol 23: 1363-1368.
17. Huizinga TW, Amos CI, van der Helm-van Mil AH, Chen W, van Gaalen FA, et al. (2005) Refining the complex rheumatoid arthritis phenotype based on specificity of the HLA-DRB1 shared epitope for antibodies to citrullinated proteins. Arthritis Rheum 52: 3433-3438.
18. Irigoyen P, Lee AT, Wener MH, Li W, Kern M, et al. (2005) Regulation of anti-cyclic citrullinated peptide antibodies in rheumatoid arthritis: contrasting effects of HLA-DR3 and the shared epitope alleles. Arthritis Rheum 52: 3813-3818.
19. Gao X, Gazit E, Livneh A, Stastny P (1991) Rheumatoid arthritis in Israeli Jews: shared sequences in the third hypervariable region of DRB1 alleles are associated with susceptibility. J Rheumatol 18: 801-803.
20. Zanelli E, Huizinga TW, Guerne PA, Vischer TL, Tiercy JM, et al. (1998) An extended HLA-DQ-DR haplotype rather than DRB1 alone contributes to RA predisposition. Immunogenetics 48: 394-401.
21. de Vries N, Tijssen H, van Riel PL, van de Putte LB (2002) Reshaping the shared epitope hypothesis: HLA-associated risk for rheumatoid arthritis is encoded by amino acid substitutions at positions 67-74 of the HLA-DRB1 molecule. Arthritis Rheum 46: 921-928.
22. Michou L, Croiseau P, Petit-Teixeira E, du Montcel ST, Lemaire I, et al. (2006) Validation of the reshaped shared epitope HLA-DRB1 classification in rheumatoid arthritis. Arthritis Res Ther 8: R79.
23. Mattey DL, Dawes PT, Gonzalez-Gay MA, Garcia-Porrua C, Thomson W, et al. (2001) HLA-DRB1 alleles encoding an aspartic acid at position 70 protect against development of rheumatoid arthritis. J Rheumatol 28: 232-239.
24. Ruiz-Morales JA, Vargas-Alarcon G, Flores-Villanueva PO, Villarreal-Garza C, Hernandez-Pacheco G, et al. (2004) HLA-DRB1 alleles encoding the "shared epitope" are associated with susceptibility to developing rheumatoid arthritis whereas HLA-DRB1 alleles encoding an aspartic acid at position 70 of the beta-chain are protective in Mexican Mestizos. Hum Immunol 65: 262-269.
25. Jawaheer D, Li W, Graham RR, Chen W, Damle A, et al. (2002) Dissecting the genetic complexity of the association between human leukocyte antigens and rheumatoid arthritis. Am J Hum Genet 71: 585-594.

26. Zanelli E, Jones G, Pascual M, Eerligh P, van der Slik AR, et al. (2001) The telomeric part of the HLA region predisposes to rheumatoid arthritis independently of the class II loci. Hum Immunol 62: 75-84.
27. Singal DP, Li J, Lei K (1999) Genetics of rheumatoid arthritis (RA): two separate regions in the major histocompatibility complex contribute to susceptibility to RA. Immunol Lett 69: 301-306.
28. Mulcahy B, Waldron-Lynch F, McDermott MF, Adams C, Amos CI, et al. (1996) Genetic variability in the tumor necrosis factor-lymphotoxin region influences susceptibility to rheumatoid arthritis. Am J Hum Genet 59: 676-683.
29. Kochi Y, Yamada R, Kobayashi K, Takahashi A, Suzuki A, et al. (2004) Analysis of single-nucleotide polymorphisms in Japanese rheumatoid arthritis patients shows additional susceptibility markers besides the classic shared epitope susceptibility sequences. Arthritis Rheum 50: 63-71.
30. Ota M, Katsuyama Y, Kimura A, Tsuchiya K, Kondo M, et al. (2001) A second susceptibility gene for developing rheumatoid arthritis in the human MHC is localized within a 70-kb interval telomeric of the TNF genes in the HLA class III region. Genomics 71: 263-270.
31. Waldron-Lynch F, Adams C, Amos C, Zhu DK, McDermott MF, et al. (2001) Tumour necrosis factor 5' promoter single nucleotide polymorphisms influence susceptibility to rheumatoid arthritis (RA) in immunogenetically defined multiplex RA families. Genes Immun 2: 82-87.
