# Supplementary material for: Defining the Role of the MHC in Autoimmunity: A Review and Pooled Analysis
Source: PLoS Genet. 2008 Apr 25;4(4):e1000024. doi: 10.1371/journal.pgen.1000024 (PMC2291482; doi:10.1371/journal.pgen.1000024)
Supplement: Text S1 — Supplementary Materials and Methods. (0.09 MB PDF) [file pgen.1000024.s009.pdf]

Text S1

Supplementary Materials and Methods

Specifically we performed PubMed searches using the keywords “HLA” or “MHC” or “TNF” or “TAP” in conjunction with each disease (for example: “MS” or “multiple sclerosis”; “SLE” or “lupus” or “systemic lupus erythematosus”; “IDDM” or “T1D” or “diabetes” or “type 1 diabetes”; “inflammatory bowel disease” or “IBD” or “Crohn’s disease” or “ulcerative colitis”; “RA” or “rheumatoid arthritis”; and “association”. This was supplemented by identifying references from Tiwari and Terasaki’s 1985 text “HLA and Disease Associations” [1]. Furthermore we drew all references from any existing reviews or meta-analyses for each disease. These studies were then triaged to remove instances where raw values were not reported. Furthermore, if the same cohort was used in multiple studies, only the most complete instance of the data was used in the meta-analysis. Only variants for which there were three independent studies examining greater than 50 cases each were included in the final analysis. Phenotype and allele frequency data were included but analyzed separately in this study. In total approximately 390 studies were included across all diseases.

Statistical methods

Briefly a perl script generated allele or phenotype-specific files from each disease-specific input file and created a shell script. The shell script referenced the relevant “BatchOR” script (BatchOR_CIallele.pl or BatchOR_CIpheno.pl – available on request) to calculate the OR and 95% CI

for each disease-specific allele or phenotype. These data were collated (Table S2) and used to create Figures 1 and 2. The OR with upper and lower 95% CI for each variant is represented by the mid-point of a diamond and whiskers respectively. The number of study cases included in the analysis for each variant is indicated by the size of the diamond. Disease-relevant haplotypes are colour-coded and a schematic representation of the classical MHC is shown beneath each figure for ease of interpretation.

Additional loci appear to provide statistically significant protection from disease (upper limit CI <1.0). However, the interpretation of the latter results is unclear as they may simply reflect under representation given that other alleles show association. We have included these results in Table S2, but will not comment on them further.

The extended LD observed at the MHC does not allow differentiation between allelic and haplotypic association in the current meta-analysis. Thus, as it is vital to consider LD when interpreting association results at the MHC, our figures display statistically significant variants on the basis of their "ancestral" also known as "conserved extended" haplotypes [2-4]. Ancestral haplotypes (AH) are large conserved stretches of MHC DNA traditionally defined and fixed at *HLA-B* in class I, the complotype: *C2*, *CFB*, *C4A*, *C4B* in class III and *HLA-DR* in class II. Such haplotypes are often designated after the *HLA-B* or *HLA-DRB1* allele they bear: for example, using the aforementioned scheme, the so-called "autoimmune" ancestral haplotype, *HLA-B**8**-SC01-DRB1*0301* is known as ancestral haplotype 8.1 (AH8.1) or a *DR3* (*DRB1*0301*)-bearing haplotype. The allelic composition of all 37 ancestral haplotypes is now assigned according to that of (predominantly homozygous) reference cell lines

held by the International Histocompatibility Workshop (IHW) (see European Bioinformatics Institute database, http://www.ebi.ac.uk/; [5-7]). Eight of these cell lines, each representing a specific MHC haplotype, have been selected for sequencing across the MHC (The MHC Haplotype Project, http://www.sanger.ac.uk/HGP/Chr6/MHC/; [8]). At present the full sequence for three of these haplotypes has been published [9,10].

It is worth considering that the majority of studies investigating the role of the MHC in disease susceptibility have been undertaken in European or white North American populations. The results of this pooled analysis will therefore be most applicable to individuals of European descent and should be interpreted with caution when considering the risk of HLA alleles in non-European populations. We have made an attempt to address this issue by commenting on ethnic differences in allelic/haplotypic association throughout the text where applicable.

REFERENCES

1. Tiwari JL, Terasaki PI (1985) HLA and Disease Associations. New York: Springer-Verlag.
2. Awdeh ZL, Raum D, Yunis EJ, Alper CA (1983) Extended HLA/complement allele haplotypes: evidence for T/t-like complex in man. Proc Natl Acad Sci U S A 80: 259-263.
3. Degli-Esposti MA, Leaver AL, Christiansen FT, Witt CS, Abraham LJ, et al. (1992) Ancestral haplotypes: conserved population MHC haplotypes. Hum Immunol 34: 242-252.
4. Degli-Esposti MA, Leelayuwat C, Daly LN, Carcassi C, Contu L, et al. (1995) Updated characterization of ancestral haplotypes using the Fourth Asia-Oceania Histocompatibility Workshop panel. Hum Immunol 44: 12-18.
5. Dorak MT, Shao W, Machulla HK, Lobashevsky ES, Tang J, et al. (2006) Conserved extended haplotypes of the major histocompatibility complex: further characterization. Genes Immun 7: 450-467.
6. Jongeneel CV, Briant L, Udalova IA, Sevin A, Nedospasov SA, et al. (1991) Extensive genetic polymorphism in the human tumor necrosis factor

region and relation to extended HLA haplotypes. Proc Natl Acad Sci U S A 88: 9717-9721.
7. Sturfelt G, Hellmer G, Truedsson L (1996) TNF microsatellites in systemic lupus erythematosus-a high frequency of the TNFabc 2-3-1 haplotype in multicase SLE families. Lupus 5: 618-622.
8. Allcock RJ, Atrazhev AM, Beck S, de Jong PJ, Elliott JF, et al. (2002) The MHC haplotype project: a resource for HLA-linked association studies. Tissue Antigens 59: 520-521.
9. Stewart CA, Horton R, Allcock RJ, Ashurst JL, Atrazhev AM, et al. (2004) Complete MHC haplotype sequencing for common disease gene mapping. Genome Res 14: 1176-1187.
10. Traherne JA, Horton R, Roberts AN, Miretti MM, Hurles ME, et al. (2006) Genetic analysis of completely sequenced disease-associated MHC haplotypes identifies shuffling of segments in recent human history. PLoS Genet 2: e9.
