# Supplementary material for: Defining the Role of the MHC in Autoimmunity: A Review and Pooled Analysis
Source: PLoS Genet. 2008 Apr 25;4(4):e1000024. doi: 10.1371/journal.pgen.1000024 (PMC2291482; doi:10.1371/journal.pgen.1000024)
Supplement: Text S2 — Nomenclature of HLA and Non-HLA Alleles. (0.09 MB PDF) [file pgen.1000024.s010.pdf]

**Text S2**

**Nomenclature of HLA and non-HLA alleles**

The nomenclature of HLA alleles can be extremely confusing. Original serological typing defined the class I antigens: *HLA-A*, *-B*, and *-C*. Serological typing of the class II locus followed later. The designation of *HLA-A* and *HLA-B* specificities is exclusive, that is, while *HLA-A1* exists, *HLA-B1* does not, reflecting the original premise that these specificities were derived from a single locus. *HLA-C* antigens were termed "*Cw*" to avoid confusion over complement protein nomenclature.

The prefix "w" or "W" (for "workshop") was also used to designate provisional antigen specificity at any locus. If the designation of such antigens became acceptably defined the prefix was omitted [1].

The advent of the MLC/MLR (mixed lymphocyte culture/reaction) in 1964 allowed the characterization of the class II locus, initially designated *HLA-D*. Antigen specificities defined by this method also carry the prefix "w". For example, the MLC defined *HLA-Dw4* specificity corresponds to the following molecular and serological (in parentheses) types: *HLA-DRB1*040101* (*DR4*), *HLA-DRB1*040102* (*DR4*), *DQA1*030101* (no serological equivalent), *DQB1*030101* (*DQ7*), *DQB1*030201* (*DQ8*)) [2]. *HLA-DP* specificities can also be defined in this way as they were originally designated by cellular techniques (e.g. *HLA-DPw1*) [3]. Thus, serological and T lymphocyte (MLC) specificities tend to encompass a number of alleles that have now been defined at the molecular level. Not all

alleles defined at the molecular level will have an equivalent serological or T lymphocyte specificity. To add to the confusion certain serotypes termed “broad” specificities are subdivided into “split specificities” or “splits”. For example, *DR3* encompasses the splits *DR17* and *DR18* (see Table S1 and http://www.anthonynolan.org.uk/HIG/lists/broad.html).

Molecular typing methods have now largely superseded serological and MLC HLA typing, resulting in a further change in nomenclature in 1987 [1]. Thus, class I and class II alleles are designated by the locus and gene name separated by a hyphen, followed an asterisk (***HLA-DRB1****). The first two digits after the asterisk defines the allele of the gene and this number frequently but not always matches the serological type (*HLA-DRB1****03***); the next two digits define the subtype of the allele (*HLA-DRB1*03***01*** or *HLA-DRB1*03***02***). A further 4 digits may follow specifying synonymous or non- synonymous amino acid changes within or outside coding sequences (http://www.anthonynolan.org.uk/HIG/lists/nomenlist.html). The presence of a null (not expressed) allele is specified by an “*N*” at the end of an allele sequence (*HLA-DRB4*0103102***N**) [1]. Null alleles of the complement genes are defined “*Q0*” (quantity zero), *C4A*Q0* for instance [4].

The *MICA* and majority of the *TNF* gene cluster polymorphisms are microsatellite designations (see Hajeer et al [5] for positions of *TNF* polymorphisms in relation to the *TNF* gene family). The *TNF* promoter polymorphisms (*TNF-308A*, *TNF-857T, TNF-863A* and *TNF-1031C*) and the *TAP2* polymorphism (*TAP2-379I*) constitute single nucleotide polymorphisms (SNPs) where the number and letter

following the gene name represents the position of the SNP from the transcriptional start site of the gene and the allele of the SNP associated with disease. *MICA* polymorphisms indicate the number of microsatellite GCT repeats (encoding alanine) in exon 5 and are designated 4, 5, 6, and 9. The 5.1 polymorphism is characterized by a single base pair insertion, which results in a premature stop codon.

## REFERENCES

1. Marsh SGE, Parham P, Barber LD (2000) The HLA FactsBook: Academic Press.
2. Marsh SG, Albert ED, Bodmer WF, Bontrop RE, Dupont B, et al. (2005) Nomenclature for factors of the HLA system, 2004. Tissue Antigens 65: 301-369.
3. Dyer PA, Martin S, Stanford RE (2000) Serological Methods in HLA Typing. In: Lechler R, Warrens A, editors. HLA in Health and Disease. Second ed: Academic Press. pp. 429-440.
4. Pickering MC, Perraudeau M, Walport MJ (2000) HLA and Systemic Vasculitides, Systemic Lupus Erythematosus and Sjogren's Syndrome. In: Lechler R, Warrens A, editors. HLA in Health and Disease. Second ed: Academic Press. pp. 327-364.
5. Hajeer AH, Hutchinson IV (2000) TNF-alpha gene polymorphism: clinical and biological implications. Microsc Res Tech 50: 216-228.
